# Reln-Dab1 pathway mitigates retinal ganglion cell apoptosis in retinal ischemia-reperfusion injury

**DOI:** 10.1038/s41419-025-07742-6

**Published:** 2025-05-29

**Authors:** Ning Xu, Zongyuan Li, Xiangwen Zeng, Yilin Jiang, Tunan Sun, Shuyu Liu, Na Li, Zhao Li, Yifei Huang, Liqiang Wang

**Affiliations:** 1 Medical School of Chinese People’s Liberation Army, Beijing, China; 2 Department of Ophthalmology, The Third Medical Center, General Hospital of Chinese People’s Liberation Army, Beijing, China; 3 State Key Laboratory of Kidney Diseases, General Hospital of Chinese People’s Liberation Army, Beijing, China; 4 School of Medicine, Nankai University, Tianjin, China

**Keywords:** Retina, Apoptosis, Stroke

## Abstract

Ischemia-reperfusion (I/R) injury is associated with a variety of retinal diseases, resulting in loss of the number of ganglion cells (RGCs), retinal structural disorders, and retinal dysfunction. The Reelin protein is an important regulator of neuronal migration and synaptogenesis, and the *Reln* signaling pathway plays an essential role in regulating the targeted projection of RGC dendrites and neuronal survival, which has not been reported in retinal I/R injury. The aim of this study was to investigate the expression, role and mechanism of *Reln* in retinal I/R injury. By establishing *Reln*-CreERT2 mTmG transgenic mice, it was observed that the expression of *Reln* initially decreased and then increased after retinal I/R injury. After supplementing exogenous Reelin protein and adeno-associated virus (AAV)-targeted regulation of *Reln* in vivo, morphological and functional experiments demonstrated its effectiveness in protecting RGCs survival, maintaining morphological integrity of the retina, and inhibiting post-injury retinal dysfunction. Furthermore, integrin β1 (Itgb1) was identified as the main receptor through which Reelin exerts neuroprotective effects while regulating retinal I/R injury repair through the Dab1-PI3K-Akt pathway. These findings provide evidence supporting *Reln* pathway’s role in maintaining retinal homeostasis and facilitating injury repair. Moreover, these findings have significant implications for identifying new targets for preventing and treating various retinal diseases.

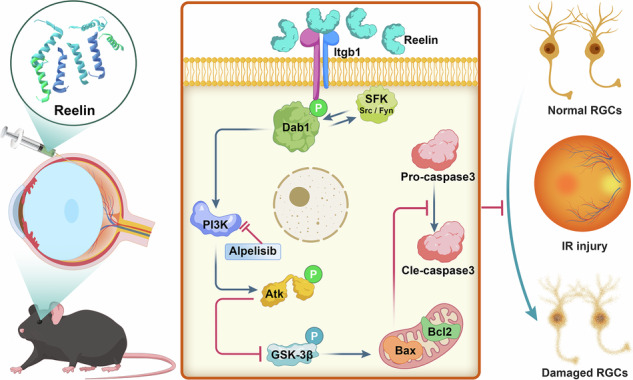

## Introduction

Retinal ischemia-reperfusion (I/R) injury is the pathological basis of a diverse spectrum of retinal diseases [1], including glaucoma, retinal vascular occlusion, diabetic retinopathy and retinopathy of prematurity [2–4]. These ocular diseases cause serious vision disorders and have a significant socio-economic impact worldwide [5]. After each episode of I/R, there is a progressive deterioration of the retinal structure and retinal cell loss, culminating in retinal ganglion cell (RGC) death and potential blindness. However, due to the lack of a clinically approved therapeutic regimen for retinal I/R injury, there is an urgent need for novel and efficacious treatment strategies [6, 7].

Obstruction of retinal blood circulation can lead to a decrease in oxygen and nutrient supply to retinal cells. Blood reperfusion induces oxidative stress, inflammation, immune response, and calcium overload in the retinal tissue, ultimately culminating in neuronal cell death [8–10]. Neuronal death can be triggered by different mechanisms, including apoptosis, iron death, and autophagy [11]. Apoptosis plays a crucial role in retinal I/R injury. It primarily operates via the mitochondria-mediated endogenous apoptosis pathway [12]. Thus, targeting the expression of key apoptotic proteins in this pathway may offer preventative and therapeutic benefits against retinal I/R damage.

Reelin, an extracellular matrix protein, is recognized for its role in promoting neuronal survival through the inhibition of apoptosis [13, 14]. Reelin-deficient mice show impaired neurogenesis and increased stroke size [15], whereas those with *Reln* gene knock-in display activation of the downstream protein target Dab1 [16]. Reelin signals via receptors such as VLDLR, ApoER2 and Itgb1 [17, 18], thereby initiating intracellular pathways through Dab1 phosphorylation and PI3K-Akt activation [19, 20]. Within the Reelin signaling pathway, Dab1 activation can trigger a cascade of events that recruit or activate various downstream factors, including Src family tyrosine kinases (SFK), which ultimately counteract neuronal apoptosis [21]. Despite these insights, the potential of Reelin to protect central neurons in the retina following I/R injury remains unexplored. This study, therefore, endeavors to elucidate the cytoprotective role of Reelin in the retinal I/R process and to dissect the underlying mechanisms.

## Materials and methods

More details are provided in the online-only Data Supplement.

### Animals

Healthy male C57BL/6J wild-type mice, aged 6–8 weeks and weighing 16–22 g, were obtained from Beijing Spefo Laboratory Animal Technology Co., Ltd, China. They were housed in a pathogen-free, temperature (22–24 °C) and humidity (40–70%) controlled environment with a 12-h light/dark cycle, and provided with water and food ad libitum.

### Generation of genetically modified mice

*Reln*-CreERT2 mTmG Mice: Generated using CRISPR-Cas9 and Cre-LoxP systems, these mice were sourced from Beijing Biocytogen Co., Ltd, China, and feature a *Reln*-CreERT2 line interbred with ROSAmT/mG for cell tracing. Initially expressing tdTomato fluorescence, Cre-mediated recombination switches expression to green fluorescent protein (GFP) in *Reln*-expressing cells. Male mice aged 6–8 weeks were used, with tamoxifen administered intraperitoneally 2 weeks prior to experiments to induce fluorescence shift.

Retinal Localized *Reln* knockdown Mice: A localized *Reln* gene knockdown was induced in C57BL/6J wild-type mice using adeno-associated virus (AAV-2/9-shRNA-*Reln*; HANBIO, Wuhan, China). Healthy males aged 6–8 weeks were injected with AAV solution (2 μL, 1.0 × 10^12^ vg/mL) into the vitreous body 3 weeks before experiments. The AAV vector encoded EGFP for tracking, with the siRNA sequence targeting *Reln* being CCAGGATACATGATGCAATTT.

### Retinal I/R model establishment

We induced retinal I/R injury in mice using a 33-gauge needle to maintain intraocular pressure (IOP) at 110 mmHg for 60 min, following a balanced salt solution protocol [22]. A sham group served as a control. After IOP normalization, mice were monitored with a heat pad and treated with tobramycin ointment (Alcon, Texas, USA) to prevent infection. Eyes were harvested at 24 h, 3, 7, 14, and 28 days post-I/R for analysis.

### Experimental groups

The experimental animals were randomly divided into three groups:

Reelin Protein Group: Received 2 μL recombinant Reelin protein (300 ng/μL; R&D Systems, Minn, USA) or PBS in the vitreous cavity 24 h pre-I/R, forming Reelin and PBS groups, with post-modeling designations as -I/R counterparts. Dosage selection of Reelin protein is shown in Fig. S1A, B.

*Reln* knockdown Group: Received 2 μL AAV-*shReln* or AAV-EGFP in the vitreous cavity 3 weeks pre-I/R, forming AAV-*shReln* and AAV-EGFP groups, with post-modeling designations as -I/R counterparts.

Alpelisib group: Mice were orally gavaged with placebo (0.5% Sodium carboxymethyl cellulose; MedChem Express, NJ, USA) or Alpelisib (50 mg/kg) 14 days prior to I/R. The treatment was administered once daily until tissue collection. This regimen was selected based on previous in vivo studies that demonstrated its efficacy and safety (Fig. S1C) [23].

### Immunohistochemistry

Mouse eyes were enucleated and fixed in a solution containing 4% paraformaldehyde at room temperature (RT) for 2 h followed by the incubation in 30% sucrose solution at 4 °C overnight and then embedded in an OCT compound. Cryosections of 5 µm-thickness were incubated with goat serum at RT for 1 h. After incubation with primary antibodies at 4 °C overnight, the sections were incubated with secondary antibodies at RT for 2 h. Antibody information is described in Table S1. Anti-fluorescence quenching sealer containing DAPI was used to seal the films (ab104139; Abcam, Cambridge, UK). Mouse eyeball sections across the optic nerve plane were selected. Images were taken with the Olympus VS200 Virtual Slide Scanning microscope (Olympus, Tokyo, Japan). Quantitative analysis of the inner retinal layer (from the internal limiting membrane to the inner edge of the outer plexiform layer) was performed using ImageJ software (National Institutes of Health, USA).

### Quantitative real-time polymerase chain reaction (RT-qPCR)

Mouse retinal tissues were processed for mRNA extraction using Trizol reagent (Invitrogen, Grand Island, NY, USA). The mRNA was subsequently reverse-transcribed into cDNA with the iScript cDNA Synthesis Kit (Bio-Rad, Hercules, CA, USA). RT-qPCR was conducted utilizing the iTaq Universal SYBR Green Supermix (Bio-Rad, Hercules, CA, USA) on a CFX96 RT-qPCR detection system. Relative gene expression was calculated using the comparative 2-ΔΔCt method, with primer sequences detailed in Supplementary Table S2.

### Hematoxylin-eosin staining (H&E)

The excised eyes were promptly fixed in 4% paraformaldehyde and subsequently embedded in paraffin. Retinal morphology was assessed through hematoxylin and eosin staining of 5-μm-thick cross-sections along the optic nerve axis. Measured the thickness of the inner retinal layer. The images were taken with a light microscope (Olympus).

### Stretched preparation of the retina

Retinas were fixed in 4% paraformaldehyde for 3 h, incised at the corneoscleral rim, and dissected to remove the cornea, iris, lens, and vitreous body. The optic nerve was separated from the sclera, and the retina was detached from the uvea. After removing pigmented tissue and rinsing in 0.1% PBST, retinas were dehydrated in methanol, blocked with goat serum, and incubated with the RGC marker RBPMS polyclonal antibody (Proteintech, Wuhan, China) overnight at 4 °C. Following secondary antibody incubation, retinas were mounted and sealed with DAPI-containing sealer. Photographic documentation and subsequent cell count analysis were conducted using a high-content imaging system (Operetta CLS, PerkinElmer, UK). The calculation of the entire retinal area was performed using ImageJ software (National Institutes of Health, USA). Antibody information is described in Table S1.

### Intravitreal injection

Anesthetized mice received intraocular injections of 2 μL recombinant Reelin protein, PBS, AAV-*shReln*, or AAV-EGFP using a 34-gauge microinjector (Hamilton, Bonaduz, Switzerland). The injection site was the superior temporal quadrant, 1 mm from the corneal limbus, with care to avoid the lens and target the optic disc. Agents were delivered gradually into the vitreous cavity.

### Optical coherence tomography (OCT)

Pupils were dilated with compound tropicamide eye drops (Santen Pharmaceutical Co., Ltd, Osaka, Japan), followed by ophthalmic surface anesthesia using oxybuprocaine hydrochloride. A carbomer-based eye drop gel (Bausch & Lomb, New York, USA) was applied to the cornea. The ophthalmic ultramicrographic imaging system (Optoprobe, Leeds, UK) was calibrated for optimal retinal imaging via OCT. Retinal thickness, measured from the internal limiting membrane to the retinal pigment epithelium, was evaluated using proprietary software (version 2.0) from OptoProbe Research Ltd.

### Flash electroretinogram (FERG)

After a 12-h dark adaptation, mice were anesthetized, immobilized and mydriasis dilated. A gold-plated wire loop, in contact with the corneal surface, served as the active electrode, while stainless-steel needle electrodes were inserted between the ears and into the caudal skin, serving as reference and ground leads, respectively. The OPTO-III visual electrophysiology instrument (Optoprobe) was operated according to its standard protocol. FERG waveform analysis involved measuring the a-wave amplitude from baseline to the first negative peak and the b-wave amplitude from the a-wave trough to the subsequent positive peak.

### Single-cell data analysis

Sequencing data for this study were obtained from the Genome Sequence Archive at the Beijing Institute of Genomics (BIG) Data Center under project PRJCA008174 (GSA registration CRA006042). Data processing was performed using CellRanger (version 7.1.0), followed by quality control and analysis with Seurat (version 4.3.0.1). Cells with over 200 detected genes and less than 20% mitochondrial gene content were included. After data merging, SCTransform normalization and PCA were conducted, with batch effects corrected using the harmony function. Clustering and visualization were based on the first 20 principal components using t-SNE. Marker genes were identified with Seurat’s FindAllMarkers function. DEG enrichment and pathway analysis were performed using the ClusterProfiler package (version 4.6.2) with the KEGG database. Single-cell data from GSE137400 were used to validate the co-expression of Reln, Itgb1, and Dab1 in RGCs, visualized using Seurat’s FeaturePlot. Protein interactions were predicted with the STRING database and visualized using Cytoscape (version 3.10.1). Intercellular communication was analyzed using the CellChat package (version 2.1.0) in R, which quantifies communication networks and predicts dominant signaling pathways. Detected ligand-receptor pairs were integrated into a protein-protein interaction network for further analysis. CellChat estimated the probabilities of cell interactions, enabling the identification of significant biological communication patterns. The intercellular signaling data were synthesized into a circular graph for visual representation.

### TUNEL labeling

Retinal cryosections were fixed, permeabilized, and subjected to TUNEL staining using the Beyotime kit (C1090; Jiangsu, China). After incubation with TDT enzyme and fluorescent labeling solution, sections were mounted with DAPI-containing medium and visualized with an Olympus VS200 microscope. Cell quantification was performed with ImageJ software (National Institutes of Health, USA).

### Western blot (WB) analysis

At 3 days post-I/R injury, retinas were harvested and lysed in RIPA buffer containing protease inhibitors. Protein extracts were quantified using a BCA assay kit (Promega, Madison, WI, USA). Proteins were separated by 10% SDS-PAGE and transferred to nitrocellulose membranes. Following a 30-min block with 5% skimmed milk, membranes were incubated with primary antibodies overnight at 4 °C, then with HRP-conjugated secondary antibodies for 2 h at RT. Immunoreactive bands were visualized using the ChemiDoc™ MP Imaging System (Bio-Rad), and band intensities were quantified with ImageJ software (National Institutes of Health, USA). Antibody information is described in Table S1.

### Statistical analysis

Sample sizes are indicated in the figure legends and were consistent with previous studies. Before data analysis, normal distribution test and variance homogeneity test were performed. All data were expressed as mean ± SD. The comparison between the two groups is involved. If the data conform to normal distribution, the *t*-test is used. If the data does not conform to the normal distribution, the nonparametric Mann–Whitney test is used. Comparisons between multiple groups (*n* ≥ 3) were involved. If the data were normally distributed, one-way ANOVA (with Dunnett’s multiple-comparisons test) was used. If the data does not conform to the normal distribution, the nonparametric Kruskal-Wallis test is used. Statistical analyses and graphs were generated using GraphPad Prism 9 statistical software (GraphPad Software, Inc., San Diego, CA, USA). Statistical significance was considered at *P* < 0.05, and all experiments were repeated at least three times.

## Results

### Characteristics of expression changes of *Reln* gene after injury

**Fig. 1:**
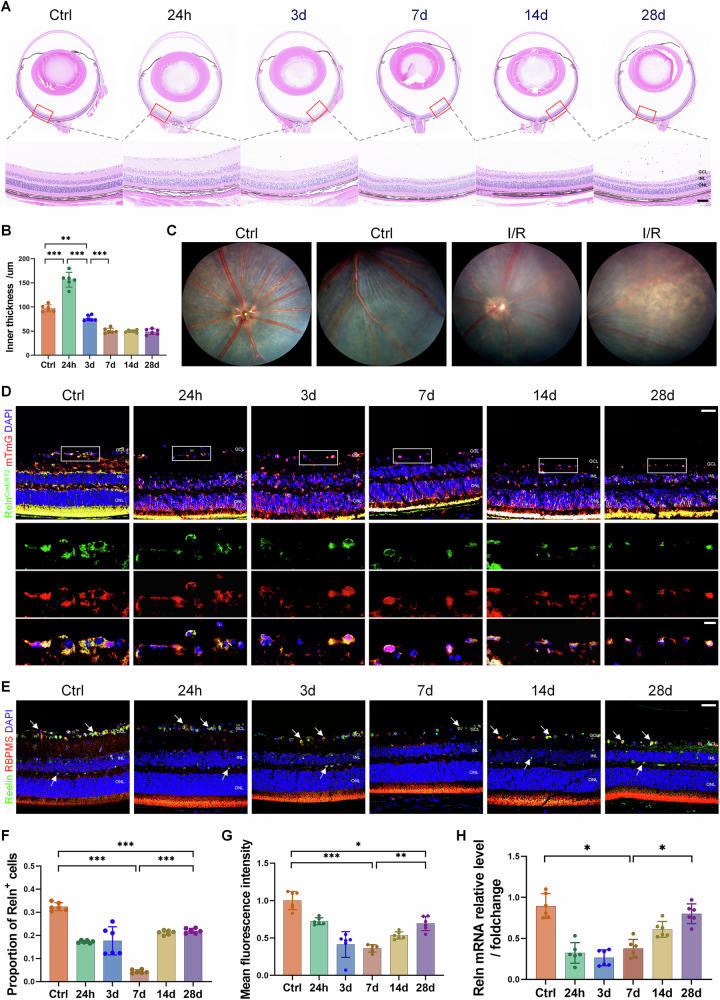
Expression of *Reln* in the retina after I/R injury. **A** Histopathological examination of retinal tissue stained with H&E showed significant edema in the inner retina layer 24 h after I/R injury, with loose and disordered cell arrangement. After 3 days, the inner retinal layer gradually became thinner, and the number of cells decreased. Scale bar: 80 μm. **B** Quantification of inner retinal thickness. **C** Fundus photography shows intermittent blood supply to retinal blood vessels after I/R injury, peripheral retinal atrophy, and visible choroid membrane. **D** *Reln*-CreERT2 mTmG mice expressed *Reln*-positive cells and their progeny at various time points after retinal I/R injury. Scale bar: 40 μm or 10 μm. **E** Representative images of Reelin immunofluorescence staining at various time points after retinal I/R injury in wild-type mice, which can confirm the expression of reelin protein in RGCs. Arrows indicate Reelin-positive cells. Scale bar: 40 μm. **F** Percentage of *Reln*-positive cells in the inner retina. **G** The expression level of Reelin protein in the inner retinal layer was assessed by the intensity of the fluorescence signals. **H** Following retinal I/R injury, the mRNA expression of the *Reln* gene initially decreased and subsequently increased, but it remained lower than the normal baseline level. Data are represented as means ± SD (*n* = 6). *P < 0.05, **P < 0.01, ***P < 0.001.

The successful establishment of a retinal I/R injury model in mice was validated through multiple assessment methods. Immediately post-I/R, pronounced corneal edema was observed (Fig. S2A), and H&E staining indicated maximal retinal thinning at 7 days, followed by stabilization (Fig. 1A, B). Fundus photography captured intermittent retinal blood flow, peripheral atrophy, and visible choroid 7 days post-I/R (Fig. 1C). Immunofluorescence revealed a gradual decline in RGC numbers, stabilizing by 7 days post-injury (Fig. S2B, C).

Utilizing *Reln*-CreERT2 mTmG mice, which exhibit permanent GFP fluorescence in *Reln*-expressing cells and their progeny post-tamoxifen induction, we assessed *Reln* gene expression under normal and I/R-injured conditions. Confocal microscopy of frozen sections demonstrated GFP signal from *Reln*-positive cells in the inner nuclear layer, inner plexiform layer, and ganglion cell layer (GCL). Analysis of fluorescence in these mice at various post-I/R time points showed a transient decrease in *Reln*-positive cells in the inner retina, nadir at 7 days, with a subsequent increase yet remaining below control levels up to 28 days post-I/R (Fig. 1D, F). This trend aligned with the results of Reelin immunofluorescence staining in wild-type mice. And the localization of Reelin protein in RGCs could be determined by observing the distribution of fluorescence signals (Figs. 1E, G, S2D). In addition, RT-qPCR confirmed a significant downregulation of *Reln* gene expression at 24 h and 3 days post-I/R, with a subsequent, albeit insufficient, recovery by 7, 14, and 28 days (Fig. 1H). These findings suggest an acute reduction in *Reln* gene expression following retinal I/R injury, implicating its role in injury response and warranting further investigation.

### Reelin protein promotes retinal tissue damage repair

**Fig. 2:**
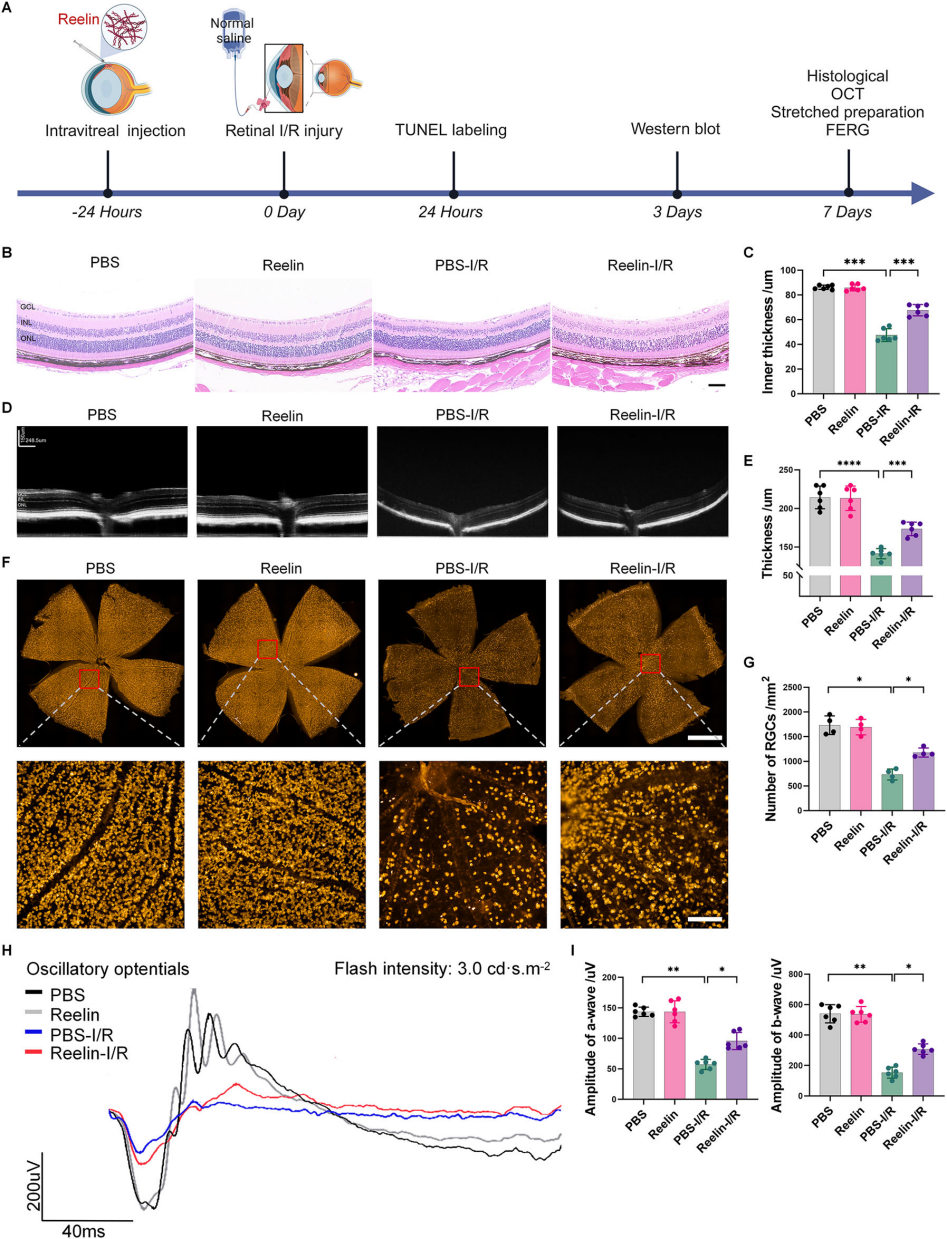
Reelin protein rescues retinal damage following I/R injury both structurally and functionally. **A** Schematic representation of the experimental design (Created with BioRender.com). **B** Histopathological examination of retinal tissue stained with H&E showed that the inner retinal thickness of mice was significantly reduced after I/R injury. This I/R-induced inner retinal thinning was significantly improved in mice treated with exogenous Reelin protein. Scale bar: 80 μm. **C** Quantification of inner retinal thickness (*n* = 6). **D** Representative OCT images of retinal tissue demonstrate changes after I/R injury. **E** Quantification of total retinal thickness (*n* = 6). **F** Representative fluorescent staining of retinal patches after I/R injury showed the RBPMS-positive RGCs (orange) distribution. Supplementation with Reelin protein exerted a significant protective effect on I/R-induced RGC injury. Scale bars: 100 μm or 1 mm. **G** Number of RBPMS-positive RGCs (*n* = 4). **H** Representative oscillatory potential waveforms of visual function were assessed via FERG 7 days after I/R injury. **I** Quantification of the magnitude of a and b waves (*n* = 6). Data are represented as means ± SD. *P < 0.05, **P < 0.01, ***P < 0.001, ****P < 0.0001.

Considering the initial downregulation of Reelin expression during the early stages of retinal I/R injury, we introduced exogenous recombinant Reelin protein into the vitreous cavity 24 h before injury induction to assess its role in retinal repair. Control mice received PBS injections (Fig. 2A). Retinal assessment at the peak damage period (7 days post-I/R) revealed significant inner retinal thinning in I/R-injured mice, which was mitigated in Reelin-supplemented mice as demonstrated by H&E staining (Fig. 2B, C). OCT further confirmed Reelin’s protective effect on overall retinal thickness post-injury (Fig. 2D, E). Immunofluorescence staining indicated a preservation of RGCs in Reelin-treated mice, evidenced by increased RGC counts compared to the PBS-I/R group (Fig. 2F, G). FERG assessments reflected a decline in a-wave and b-wave amplitudes post-I/R injury, which were significantly improved in the Reelin-I/R group (Fig. 2H, I). Moreover, the b-wave amplitude was slightly increased in the uninjured Reelin group compared to the PBS group, but this difference did not exhibit statistical significance. In conclusion, the supplementation of Reelin protein demonstrated therapeutic potential in facilitating retinal repair and may serve as a promising intervention for retinal ischemic diseases.

### *Reln* signaling pathway regulates retinal tissue damage repair

**Fig. 3:**
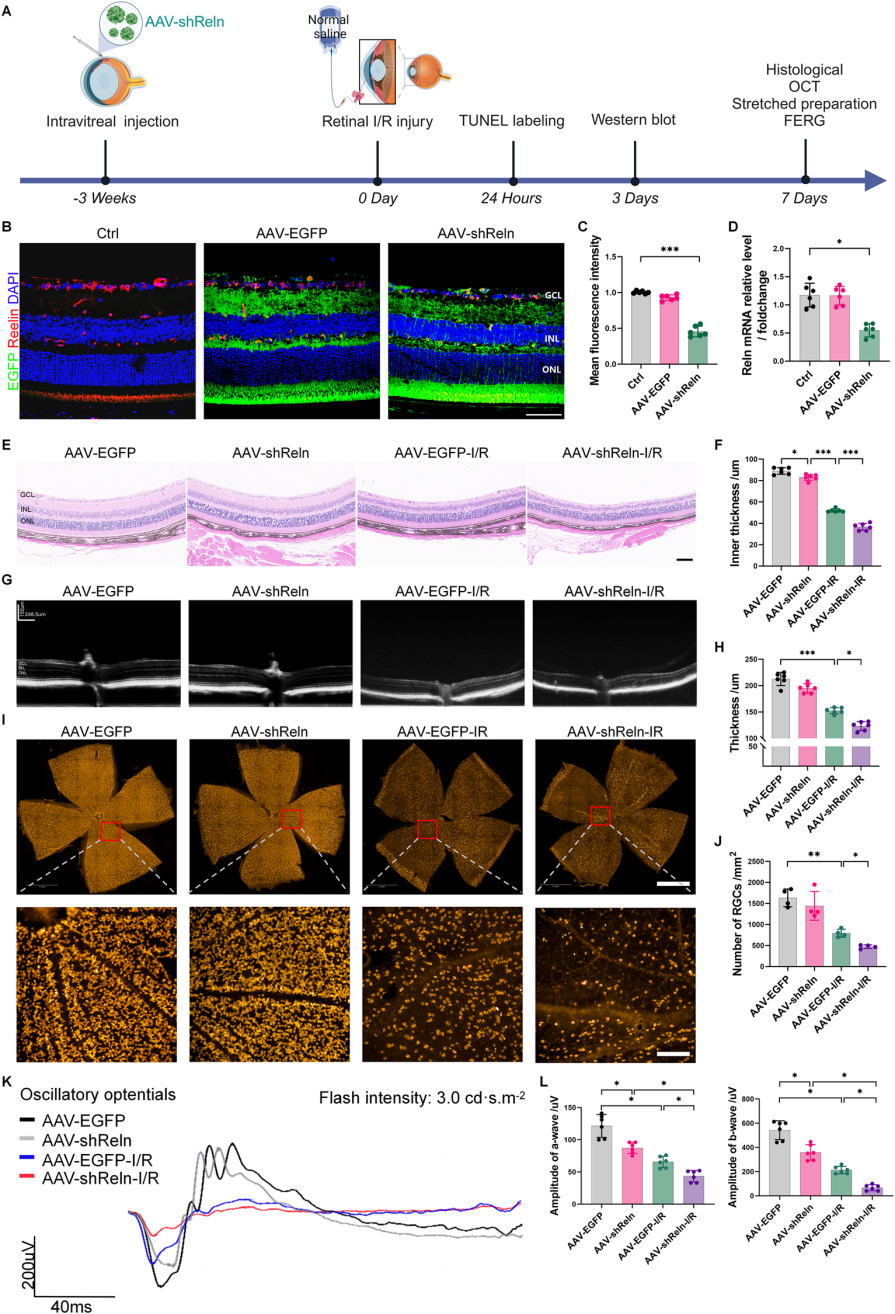
AAV-*shReln*-mediated local knockdown of *Reln* aggravated retinal thickness thinning, the reduction in RGC count, and retinal dysfunction in mice caused by I/R injury. **A** Schematic representation of the experimental design (Created with BioRender.com). **B** Frozen slides showing retinal infection 3 weeks after intravitreal injection of AAV. EGFP carried by AAV was expressed in nearly all the layers of the retina. Scale bar: 40 μm. **C** The expression level of Reelin protein in the inner retinal layer was assessed by the intensity of the fluorescence signals (*n* = 6). **D** The mRNA expression of the *Reln* gene (*n* = 6). **E** Histopathological examination of retinal tissue via H&E staining showed that the inner retinal thickness in AAV-*shReln* mice was further reduced after I/R injury. The damage to I/R-induced RGCs was further aggravated in AAV-*shReln* mice. Scale bar: 80 μm. **F** Quantification of inner retinal thickness (*n* = 6). **G** Representative OCT images of retinal tissue changes after I/R injury. **H** Quantification of total retinal thickness (*n* = 6). **I** Representative fluorescent staining of retinal patches after I/R injury showed the distribution of RBPMS-positive RGCs (orange). Scale bars: 100 μm or 1 mm. **J** Number of RBPMS-positive RGCs (*n* = 4). **K** Representative oscillatory potential waveforms of visual function were assessed by FERG after I/R injury. **L** Quantification of the magnitude of a and b waves (*n* = 6). Data are represented as means ± SD. *P < 0.05, **P < 0.01, ***P < 0.001.

To investigate the *Reln* signaling pathway’s role in I/R injury, we developed a mouse model with localized retinal *Reln* knockdown. We employed AAV-mediated intravitreal injection to locally knock down the retinal *Reln* gene. Three weeks post-injection, immunofluorescence staining and RT-qPCR confirmed effective knockdown of *Reln* expression in retinal tissues (Fig. 3A, B). The knockdown model was validated (Fig. 3C, D), and the impact of I/R injury was assessed at 7 days post-injury.

H&E staining at this time point revealed a significant decrease in inner retinal thickness in I/R-injured mice, which was exacerbated in the *Reln* knockdown group (Fig. 3E, F). OCT confirmed a reduction in overall retinal thickness post-injury, particularly in *Reln* knockdown mice (Fig. 3G, H). Immunofluorescence staining demonstrated a decreased RGC density and count in the AAV-*shReln*-I/R group compared to the AAV-EGFP-I/R group (Fig. 3I, J). Although a minor decrease in retinal thickness and RGC count was observed in the uninjured AAV-*shReln* group, it was not statistically significant. Functional assessment using FERG showed a significant reduction in a and b wave amplitudes in the AAV-*shReln*-I/R group compared to the AAV-EGFP-I/R group. Interestingly, a-wave and b-wave amplitudes were also reduced in the uninjured AAV-*shReln* group, indicating a role for *Reln* in maintaining retinal function (Fig. 3K, L). Collectively, these results underscore the *Reln* pathway’s regulatory function in retinal homeostasis and its protective role in I/R injury.

### *Reln* is primarily expressed in RGCs and cone bipolar cells (CBCs) and participates in the PI3K-Akt pathway after I/R injury

**Fig. 4:**
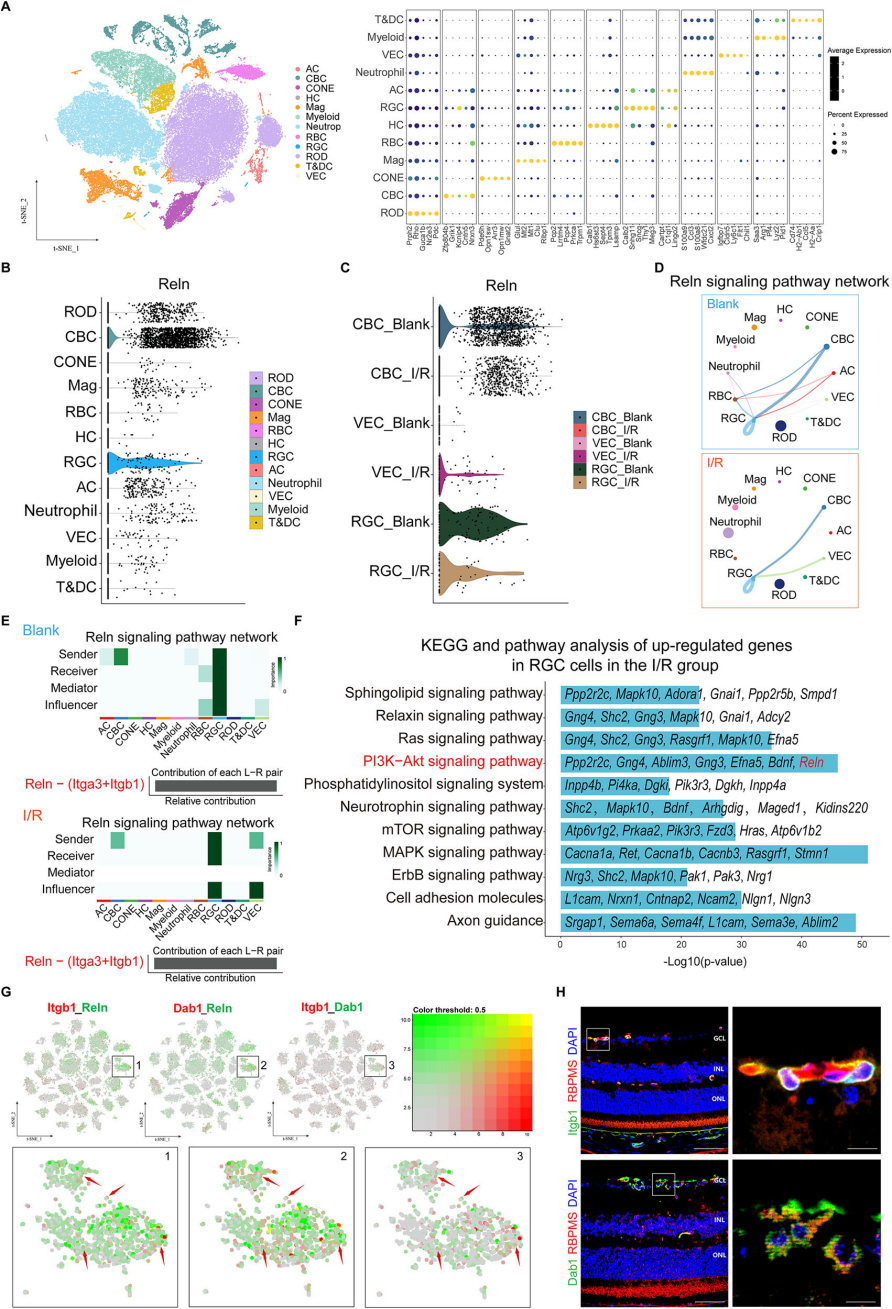
*Reln* was enriched in the PI3K-Akt pathway in RGC cells of the I/R group. **A** A dot plot based on the marker genes of each cluster (right). The t-SNE map shows 12 distinct clusters of retinal cells, each colored dot represents a cell. The size of each circle in the dot plot represents the proportion of gene expression, and the color represents the level of expression. **B** Violin Plot shows *Reln* gene expression in 12 different cell clusters. **C** Violin Plot shows *Reln* gene expression in RGC, CBC, and VEC clusters in the blank and I/R groups. **D** Circle chart shows the differences in the inferred *Reln* signaling networks between the blank and I/R groups. **E** Heatmap shows the role and importance of each cluster in the *Reln* signaling network (top). Bar charts show the relative contribution of each ligand-receptor pair in the *Reln* signaling pathway (bottom). **F** KEGG pathway enrichment analysis of RGCs in the I/R group (*P* < 0.05). **G** t-SNE charts show the expression of the *Reln*, *Itgb1* and *Dab1* genes in different RGC cell clusters, with the color representing the expression level. **H** Representative images of Itgb1 and Dab1 immunofluorescence staining in normal mice retinas.

Single-cell transcriptomic analysis was performed on retinal tissues from mice in the blank group and mice subjected to injury 3 days after retinal I/R. Based on the expression profiles of maturation markers, we identified 12 distinct cell clusters: rods, CBCs, cones, macroglia, rod bipolar cells (RBCs), horizontal cells, RGCs, amacrine cells, neutrophils, vascular endothelial cells (VECs), microglia (myeloid), T cells, and dendritic cells (Fig. 4A). The *Reln* gene was predominantly expressed in retinal RGCs and CBCs, and its overall expression level decreased after injury (Figs. 4B, S3A). Furthermore, the expression of the *Reln* gene decreased in RGCs and CBCs following I/R injury, while it increased in VECs (Fig. 4C). Subsequently, we investigated the impact of I/R on cellular interactions and found that RGCs, CBCs, and VECs were the primary sources of ligands in the *Reln* signaling network, operating in both autocrine and paracrine modes. I/R led to a reduction in the *Reln* pathway gene expression between RGCs and CBCs and increased expression between RGCs and VECs (Fig. 4D). In addition, the extracellular matrix receptor, Integrin α3β1, is posited as a potential Reelin protein target in RGCs (Fig. 4E). KEGG pathway analysis indicates that the *Reln* gene is primarily involved in the PI3K-Akt pathway, which is associated with the heightened activities in pathways of responding to extracellular signals, promoting metabolism, proliferation, and cell survival following I/R injury (Fig. 4F). Afterward, we analyzed the interactions between Dab1, a key factor in the *Reln* pathway, and the related proteins mentioned above. The protein-protein interaction (PPI) network showed that there can be interactions between Reln, Itgb1, and Dab1, and both Itgb1 and Dab1 can activate the PI3K-Akt pathway (Fig. S3B). Due to the small number of RGCs in this dataset, expanded analysis utilizing RGC single-cell transcriptome data identified 45 molecular clusters [24] with widespread *Reln* expression and co-expression of *Itgb1*, *Dab1*, and *Reln* (Figs. S3C, 4G). And we verified the expression of Itgb1 and Dab1 in RGCs by immunofluorescence (Fig. 4H).

In addition, we confirmed, through immunofluorescence staining, that the *Reln* gene was primarily expressed in CBCs rather than RBCs (Fig. S3D). CBCs could be further classified into three subclusters (SCs), with differential subcluster expression patterns observed between control and I/R groups (Fig. S3E–G). A novel subcluster, CBC-SC2, emerged post-I/R, potentially contributing to explaining the increase in *Reln*-positive cells in later stages of injury (Fig. S3H). These results collectively indicate that the *Reln* gene, predominantly expressed in RGCs and CBCs, is involved in the PI3K-Akt signaling pathway, and its dysregulation post-I/R injury may disrupt retinal cell signaling, leading to associated retinal tissue damage.

### Reelin protein inhibits apoptosis after I/R injury through the Dab1-PI3K-Akt pathway

**Fig. 5:**
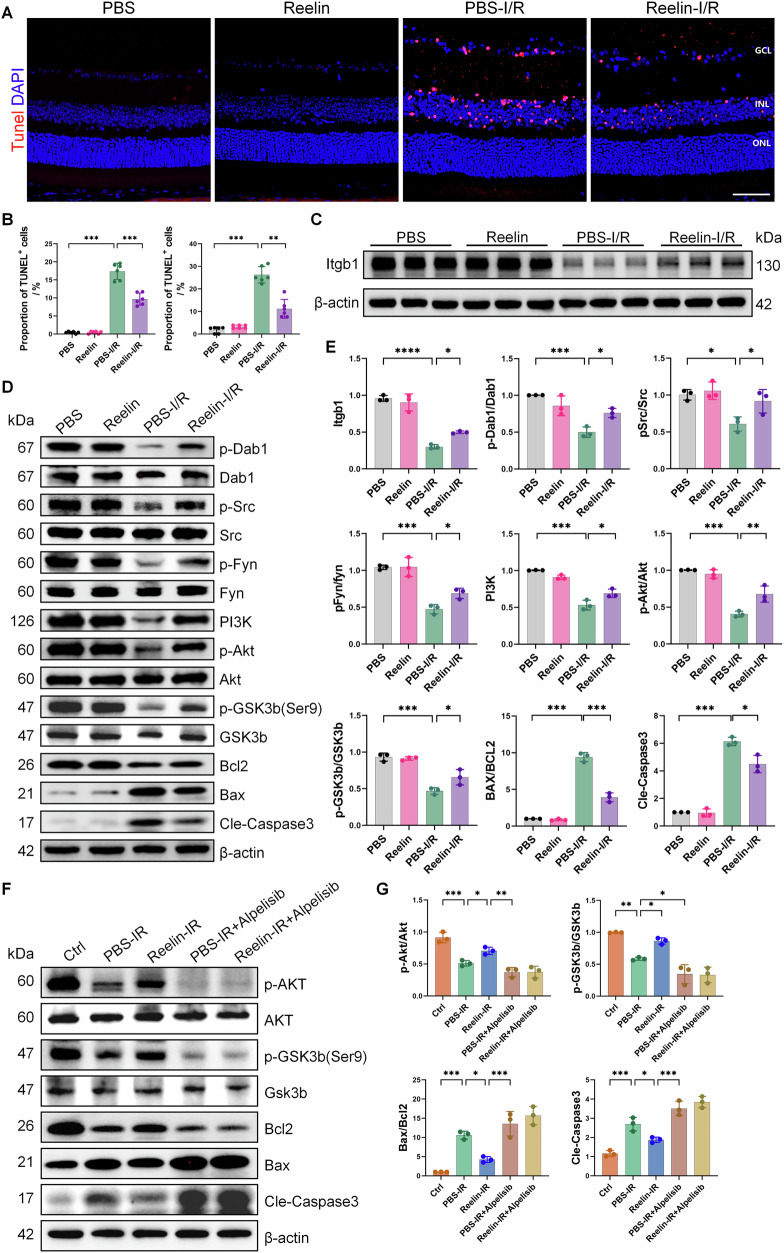
After retinal I/R injury, Reelin protein inhibits apoptosis through the Dab1-PI3K-Akt pathway. **A** Representative TUNEL staining of frozen retinal sections 24 h after I/R injury. Scale bar: 40 μm. **B** Quantification of TUNEL-positive cells in the GCL (left) and inner retinal layer (right), (*n* = 6). **C**, **D** Representative blot images of the effects of Reelin protein therapy on retinal Itgb1, Src, p-Src, Fyn, p-Fyn, Dab1, p-Dab1, PI3K, Akt, p-Akt, GSK3b, p-GSK3b, Bcl-2, Bax and Cle-Caspase3 after I/R injury. **E** Quantitative analysis of the levels of the aforementioned proteins. **F** Representative blot images showing the effects of PI3K inhibitors on Akt, p-Akt, GSK3b, p-GSK3b, Bcl-2, Bax, and Cle-Caspase3 in the retina following I/R injury treated with Reelin protein. **G** Quantitative analysis of the levels of the aforementioned proteins (*n* = 3). Data are represented as the means ± SD. *P < 0.05, **P < 0.01, ***P < 0.001, ****P < 0.0001.

As previously demonstrated, Reelin protein reduced RGC death in the mouse retina post-I/R (Fig. 2F, G), and single-cell sequencing indicated activation of the PI3K-Akt pathway following I/R injury (Fig. 4F). To explore the mechanism of Reelin-induced neuroprotection, we evaluated its impact on apoptosis via TUNEL staining (Fig. 5A). TUNEL staining revealed peak retinal cell apoptosis at 24 h post-I/R (Fig. S4A, B). In contrast to the negligible TUNEL-positive cells in the uninjured group, a significant increase was observed 24 h post-I/R, with a substantial decrease in TUNEL-positive cells in both the GCL and inner retina in the Reelin-I/R group (Fig. 5B). This suggests that Reelin effectively attenuates retinal cell apoptosis post-injury.

To further explore the anti-apoptotic mechanism of Reelin protein, combined with single-cell transcriptome analysis results (Fig. 4E, F), we performed WB analysis of the samples, revealing increased Itgb1 expression post-injury in the presence of exogenous Reelin (Fig. 5C, E). Activation of Fyn, Src, Dab1 and PI3K was observed in the Reelin-I/R group, with no change in Akt and GSK3b levels, but a notable increase in phosphorylated proteins p-Akt and p-GSK3b(Ser9) compared to the PBS-I/R group. Additionally, the Reelin-I/R group exhibited significantly lower expression of apoptosis-related protein Caspase-3 and a reduced Bax/Bcl-2 ratio (Fig. 5D, E). Collectively, these findings indicate that Reelin protein supplementation post-retinal I/R injury is neuroprotective, promoting Dab1 phosphorylation, activating the PI3K-Akt-GSK3b pathway, and inhibiting retinal cell apoptosis. In addition, we performed a detailed analysis of the protein expression levels of p35 and p25, two key regulatory proteins of cyclin-dependent kinase 5 (Cdk5). Our results indicated that I/R injury significantly increased the P25/P35 ratio, suggesting the aberrant activation of the Cdk5 pathway. However, Reelin protein failed to counteract this increase. This suggests that under I/R-induced injury conditions, Reelin did not exert repair or regulatory functions via the Cdk5 pathway (Fig. S5A, B).

### The PI3K inhibitor Alpelisib reverses the anti-apoptotic effect of Reelin protein

To further elucidate whether Reelin protects retinal cells from I/R-induced damage via the PI3K-Akt pathway, the PI3K inhibitor Alpelisib was administered orally to mice, and its effects on Reelin-mediated protection of retinal cells against I/R-induced damage were investigated. Our study revealed that Alpelisib administration significantly downregulated the expression of p-Akt protein (Fig. 5F, G), thereby confirming its inhibitory effect on the PI3K-Akt pathway. Furthermore, Alpelisib reversed the inhibitory effects of Reelin protein on I/R-induced pro-apoptotic factors, including GSK3b, Bax, and Cleaved-Caspase3. Conversely, the expression of the anti-apoptotic protein Bcl-2 was significantly reduced (Fig. 5F, G). These findings demonstrate that Alpelisib administration markedly diminished the protective effects of Reelin protein on I/R-induced retinal cells and promoted cell apoptosis. Collectively, these results further support the notion that Reelin protein exerts its protective role against I/R-induced retinal cell damage by activating the PI3K-Akt pathway.

### *Reln* deficiency promotes apoptosis through the Dab1-PI3K-Akt pathway

**Fig. 6:**
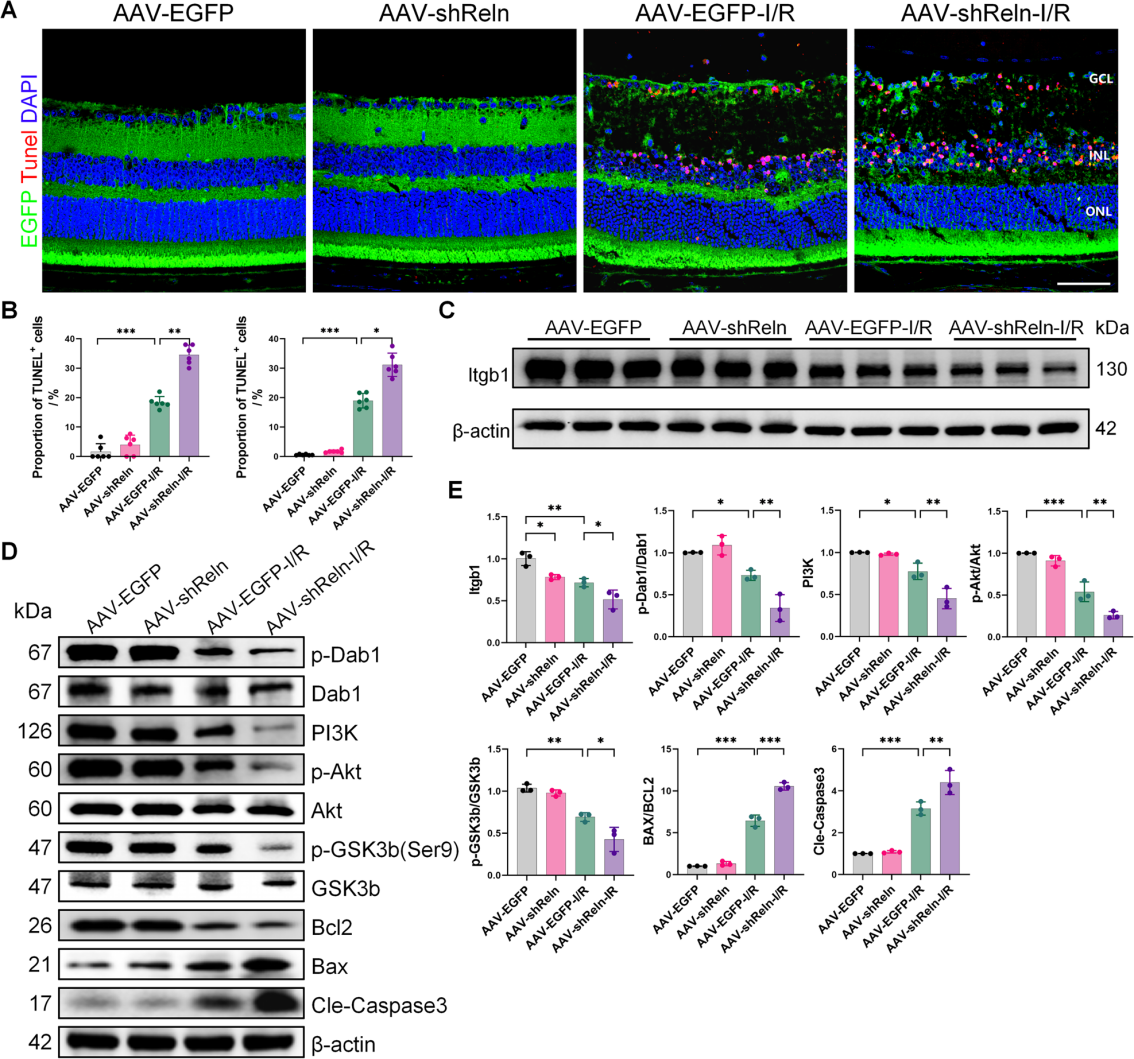
Local knockdown of *Reln* promotes apoptosis after I/R through the Dab1-PI3K-Akt pathway. **A** Representative TUNEL staining of frozen retinal sections of AAV-*shReln* mice 24 h after I/R injury. Scale bar: 40 μm. **B** Quantification of TUNEL-positive cells in the GCL (left) and inner retinal layer (right), (*n* = 6). **C**, **D** Representative blot images of the effects of *Reln* local knockdown on retinal Dab1, p-Dab1, PI3K, Akt, p-Akt, GSK3b, p-GSK3b, Bcl-2, Bax, cle-caspase3 and Itgb1 after I/R injury. **E** Quantitative analysis of the levels of the aforementioned proteins (*n* = 3). Data are represented as the means ± SD. *P < 0.05, **P < 0.01, ***P < 0.001.

As previously shown, *Reln* gene knockdown intensifies RGC loss in the mouse retina post-I/R injury (Fig. 3I, J). To explore the mechanism by which the *Reln* signaling pathway influences I/R injury, we examined the effect of *Reln* knockdown on apoptosis using TUNEL staining (Fig. 6A). Consistent with prior findings, TUNEL-positive cells were negligible in the uninjured group but markedly increased 24 h post-I/R injury, with an exacerbated increase in the context of *Reln* gene knockdown (Fig. 6B). These observations indicate that *Reln* knockdown exacerbates apoptosis following I/R injury.

Further mechanistic investigation via WB analysis disclosed no significant changes in Dab1, Akt and GSK3b expression levels in the AAV-*shReln*-I/R group. However, expression of PI3K and phosphorylated proteins p-Dab1, p-Akt and p-GSK3b(Ser9) was significantly reduced compared to the AAV-EGFP-I/R group. Additionally, the expression of apoptotic markers, Caspase-3 and the Bax/Bcl-2 ratio, was significantly elevated in the AAV-*shReln*-I/R group. Notably, Itgb1 protein expression was lower in the AAV-*shReln*-I/R group than in the AAV-EGFP-I/R group (Fig. 6C–E). In summary, *Reln* gene knockdown results in reduced Dab1 phosphorylation and impedes the activation of the PI3K-Akt-GSK3b pathway following retinal I/R injury, thereby promoting retinal cell apoptosis post-injury.

## Discussion

In this study, we demonstrate for the first time the neuroprotective role of Reelin protein in promoting the survival of retinal neurons, a significant advancement in addressing retinal ischemia. Retinal ischemia-reperfusion, implicated in the pathogenesis of glaucoma, is characterized by increased IOP and RGC death, leading to irreversible vision impairment globally [25]. Current treatments, including eye drops, intraocular injections (e.g., anti-VEGF), and surgery, aim to halt disease progression but have inherent limitations, prompting the search for safer and more effective alternatives. We found basal Reelin expression in normal mouse retinas, with a decrease during the acute phase of I/R injury. Prophylactic administration of exogenous recombinant Reelin protein was observed to significantly reduce RGC loss, retinal thinning, and partially ameliorate visual function impairments. Conversely, *Reln* gene knockdown exacerbated RGC death and visual dysfunction post-I/R injury. Mechanistic insights reveal that Reelin’s neuroprotection is mediated through the activation of the Dab1-PI3K-Akt pathway, underscoring its potential as a therapeutic target for retinal ischemic diseases.

Previous research indicates that Reelin expression peaks during retinal development, essential for the migration, positioning, and differentiation of retinal cells—a physiological upregulation [26]. In adulthood, Reelin expression subsides to baseline, only to re-emerge in response to injury or degeneration [27]. To monitor cells expressing *Reln* and their progeny in the retina, we developed the *Reln*-CreERT2 mTmG transgenic mouse model. Following tamoxifen induction, the tomato sequence is excised by Cre recombinase, and a membrane-tagged green fluorescent protein is expressed, as previously described [28]. Our study tracked *Reln* expression post-retinal I/R injury, noting an initial decline in both *Reln* gene expression and protein levels, followed by an increase that still fell short of baseline levels. The early decrease in Reelin post-I/R may reflect a reduction in Reelin-expressing RGCs or CBCs due to injury. Conversely, the late-stage increase may result from the activation of Reelin expression in VECs, CBC-SC2, and blood or stromal cells, indicative of an injury-repair response aimed at maintaining retinal integrity.

Existing literature on Reelin and I/R injury has predominantly focused on the central nervous system. In vitro studies have reported that recombinant Reelin protein confers significant neuroprotection against oxidative stress in neurons. Corresponding in vivo experiments have illustrated that pre-administration of exogenous Reelin protein into the ventricles prior to middle cerebral artery occlusion reduces cerebral infarct volume and promotes neuronal survival [13, 14]. Our study pioneers the investigation of the neuroprotective effects of exogenous Reelin protein on the retina. Our study selected a well-established model of in vivo induced retinal I/R injury in which the entire retina is induced to ischemia, resulting in damage to multiple retinal cell types, primarily RGCs [11]. The retina demonstrates distinct sensitivity to ischemia in different regions, with the outer layer exhibiting less sensitivity than the inner layer [29]. Therefore, our research mainly focuses on the inner layer of the retina. Our findings underscore the role of the *Reln* gene and Reelin protein in preserving the inner retinal layer’s morphology and the retina’s overall structural integrity during I/R. The principal cellular damage post-retinal I/R injury is characterized by a decline in RGC count, which is crucial for visual information transmission to the brain. Notably, Reelin protein provides substantial protection for RGC survival.

Additionally, we utilized FERG to evaluate visual function, revealing a significant positive correlation between the reduction in FERG amplitude and the deterioration of visual function post-I/R injury. This correlation is indicative of compromised electrophysiological function among retinal neurons. FERG parameters, notably the Max wave, offer an overview of general retinal functionality, while the a and b waves specifically reflect the status of the outer and inner retina, respectively. Our study demonstrated that Reelin treatment partially restored the amplitudes of both a-wave and b-wave in I/R-injured mice, signifying an amelioration of retinal dysfunction. Consequently, exogenous Reelin protein not only fosters neuronal survival and preserves retinal morphology but also significantly enhances retinal function in mice following I/R injury.

To further validate the pivotal role of the *Reln* signaling pathway in retinal I/R injury, we examined the consequences of I/R-induced retinal injury in AAV-*shReln* mice. Given the survival challenges and neurological abnormalities associated with *Reln* deficiency in Reeler mice [30], we opted for a localized retinal *Reln* knockdown model using AAV, which offers high target specificity alongside low carcinogenicity and immunological risk [31]. We found that *Reln* knockdown resulted in a more pronounced thinning of both the inner and overall retinal layers following injury, suggesting a more severe alteration in retinal tissue morphology in retinal tissue. Strikingly, the inner retinal layer in uninjured *Reln* knockdown mice already displayed significant thinning, whereas the overall retinal thinning, while present, was not statistically significant, underscoring the *Reln* gene’s pronounced influence on the inner retinal layer in the uninjured state. Furthermore, a more pronounced reduction in RGC count and increased visual dysfunction were observed in *Reln* knockdown mice following I/R injury, with even uninjured knockdown mice demonstrating significant visual impairment. These outcomes not only emphasize the regulatory role of the *Reln* signaling pathway in maintaining normal retinal homeostasis but also further validate its neuroprotective role in promoting injury repair.

Previous studies have identified apoptosis as a prominent mechanism of injury in I/R-associated retinopathy [32]. We aimed to further investigate the regulatory role of the *Reln* gene and the Reelin protein in RGC apoptosis through TUNEL staining. Additionally, we quantified key proteins involved in the mitochondrial apoptotic pathway via WB analysis to elucidate the potential mechanisms underlying Reelin’s contribution to I/R injury. The Bcl-2 protein family, which represents the first documented group of apoptosis-related proteins, is known to play a key role in mediating mitochondrial apoptosis [33]. Numerous studies have demonstrated that Bcl-2 and Bax are major regulators in the Caspase-3-dependent apoptotic pathway that determines neuronal fate. In the context of retinal I/R injury, Caspase-3 is regarded as the primary executor of apoptosis in the inner layer of the rodent retina [34]. Our findings indicate an increase in Caspase-3 protein expression and the Bax/Bcl-2 ratio following I/R injury. However, pretreatment with Reelin protein led to a decrease in both metrics, which in turn increased following *Reln* knockdown. These results corroborate the neuroprotective mechanism of Reelin in attenuating RGC apoptosis.

Classical Reelin pathway triggers include the VLDLR, ApoER2 and Itgb1 [17, 18]. In our study, single-cell transcriptomic analysis revealed that Reln-Itgα3β1 ligand-receptor pair is a signaling driver of the *Reln* signaling pathway. Integrin, a heterodimeric cell surface receptor for extracellular matrix proteins, comprises α and β subunits, with Itgb1 being the largest integrin subfamily involved in various cellular processes, including receptor-mediated activity, cell signaling, and defense mechanisms [35, 36]. Previous study has found that Itgb1 can be activated by Reelin and induce the recruitment and activation of SFK [37]. Our findings indicate that exogenous Reelin protein significantly upregulates Itgb1 expression in retinal tissues post-I/R injury, and its expression is suppressed following *Reln* knockdown, suggesting that Itgb1 is an important signaling molecule in the *Reln* signaling pathway in the retina. Additionally, previous studies have demonstrated that SFK, particularly Fyn and Src, function as the primary physiological kinases for Dab1 and are activated by receptor clustering during Reelin signaling. Their activity is subsequently upregulated in a Dab1-dependent manner through a positive feedback mechanism [21, 38, 39]. Our findings reveal that Reelin protein robustly enhances the phosphorylation of Fyn and Src, thereby indicating the activation of the SFK signaling pathway. In vitro studies demonstrate that Reelin activates the PI3K-Akt pathway in a manner that is dependent on SFK activity and Dab1 phosphorylation. Furthermore, the activated Akt phosphorylates Ser9 of GSK3b, leading to the inhibition of GSK3b activity and the promotion of cell survival [40, 41]. In this study, we found that the Reelin protein significantly enhances Dab1 phosphorylation after retinal I/R injury, suggesting the activation of *Reln* signaling pathway. Phosphorylated Dab1 further activates the PI3K-Akt pathway, Akt phosphorylation at serine 473 is recognized for its neuroprotective effects, with p-Akt typically maintained at high levels in stable cells [42]. However, Akt phosphorylation is significantly reduced post-I/R injury. Our study demonstrates that Reelin protein promotes Akt phosphorylation, a critical process for sustaining cell survival after ischemic retinal injury. In addition, we found that PI3K expression increases in Reelin protein-pretreated mice following retinal I/R. The activation of the PI3K-Akt pathway induces phosphorylation of GSK3b at the Ser9 residue, thereby inhibiting its activity and subsequently reducing neuronal apoptosis. Conversely, the PI3K inhibitor Alpelisib effectively reverses the anti-apoptotic effects of Reelin mediated through the PI3K-Akt pathway. In addition to the aforementioned findings, our results also revealed that although I/R injury led to an increase in the P25/P35 ratio, the administration of exogenous Reelin protein did not reverse this elevation. Both Cdk5 and the Reelin pathway target tau protein as downstream substrate. The overphosphorylation of tau protein is a critical hallmark of various neurodegenerative diseases [43]. We speculate that this outcome may stem from Reelin and Cdk5 functioning in parallel rather than through a simple linear interaction [44]. In contrast, *Reln* knockdown mice exhibit contrasting trends. *Reln* knockdown resulted in marked attenuation of Dab1 phosphorylation and a marked inhibition of the PI3K-Akt pathway in the mouse retina, thereby activating GSK3b and inducing cell apoptosis. These findings substantiate that Reelin signaling activates the intracellular junction protein Dab1 via membrane receptors Itgb1, and subsequent activation of the PI3K-Akt signaling pathway. Activated p-Akt inhibits GSK3b activity and modulates the expression of Bcl-2 and Bax, as well as caspase-3, thereby inhibiting apoptosis and offering neuroprotection.

Our findings reveal that the Reln-Dab1 signaling pathway is a critical regulatory and therapeutic target in the context of retinal I/R injury, exerting its effects through the modulation of neuronal apoptosis. With the novel introduction of this pathway into the study of retinal I/R injury, we enhance the understanding of disease mechanisms. Furthermore, our findings contribute to the development of targeted therapies with potential applications in glaucoma and other I/R-related retinal conditions.

## Supplementary information

Supplementary Materials

Figure S1

figureS2

figureS3

figureS4

Figure S5

Full and uncropped western blots

Table of DEGs between Blank and IR groups in single-cell sequencing

## Data Availability

The datasets used and/or analyzed during the current study are included in this published article or are available from the corresponding author on reasonable request. Publicly available datasets were obtained from the following repositories: China National Center for Bioinformation (CRA006042) and Gene Expression Omnibus (GSE137400).
