# Supplementary material for: Reln-Dab1 pathway mitigates retinal ganglion cell apoptosis in retinal ischemia-reperfusion injury
Source: Cell Death Dis. 2025 May 29;16(1):423. doi: 10.1038/s41419-025-07742-6 (PMC12122947; doi:10.1038/s41419-025-07742-6)
Supplement: Supplementary file 1 — Supplementary Materials [file 41419_2025_7742_MOESM1_ESM.doc]

Supplementary Materials for

- Reln-Dab1 pathway mitigates retinal ganglion cell apoptosis in retinal ischemia-reperfusion injury

Ning Xu, Zongyuan Li, Xiangwen Zeng *et al.*

*Corresponding author. Email: liqiangw301@163.com (L.W.); 301yk@sina.com(Y.H.).

**This PDF file includes:**

Table S1 and S2

Figs. S1 to S5

## **Table S1. Primary and secondary antibodies used for immunohistochemical staining and western blot**

| **Target antigen** | **Vendor or Source** | **Catalog #** | **Working concentration** |
| --- | --- | --- | --- |
| Reelin | Thermo Fisher Scientific | PA5-78413 | 1/500 (IF) |
| Reelin | Abcam | ab78540 | 1/500 (IF) |
| RBPMS | Proteintech | 15187-1-AP | 1/200 (IF) |
| RBPMS | santa cruz biotechnology | sc-293285 | 1/50 (IF) |
| Dab1 | Abcam | ab111684 | 1/500 (IF) |
| Itgb1 | Abcam | ab179471 | 1/500 (IF) |
| Isl1 | Abcam | ab109517 | 1/50 (IF) |
| PKCα | Thermo Fisher Scientific | MA1-157 | 1/50 (IF) |
| Alexa Fluor 488-conjugated  Goat anti-mouse antibody | Thermo Fisher Scientific | A-11029 | 1/500 (IF) |
| Alexa Fluor 568-conjugated  Goat anti-rabbit antibody | Thermo Fisher Scientific | A-11011 | 1/500 (IF) |
| Alexa Fluor 488-conjugated  donkey anti-rabbit antibody | Thermo Fisher Scientific | A-21206 | 1:500 (IF) |
| Alexa Fluor 568-conjugated  Goat anti-mouse antibody | Thermo Fisher Scientific | A-11004 | 1/500 (IF) |
| Itgb1 | Abcam | ab52971 | 1/1000 (WB) |
| Dab1 | Abcam | ab111684 | 1/1000 (WB) |
| phospho-Dab1 | Abcam | ab78200 | 1/500 (WB) |
| Fyn | Abcam | ab125016 | 1/1000 (WB) |
| phospho-Fyn | Thermo Fisher Scientific | PA5-104756 | 1/1000 (WB) |
| Src | Abcam | ab109381 | 1/1000 (WB) |
| phospho-Src | Abcam | ab185617 | 1/1000 (WB) |
| PI3K | Abcam | ab302958 | 1/1000 (WB) |
| phospho-Akt | Cell Signaling Technology | 4060S | 1/2000 (WB) |
| Akt | Abcam | ab8805 | 1/1000 (WB) |
| GSK3B | Proteintech | 67329-1-Ig | 1/1000 (WB) |
| phospho-GSK3B (Ser9) | Thermo Fisher Scientific | MA5-14873 | 1/1000 (WB) |
| Bcl-2 | Abcam | ab182858 | 1/2000 (WB) |
| Bax | Abcam | ab32503 | 1/2000 (WB) |
| cleaved Caspase-3 | Cell Signaling Technology | 9661S | 1/1000 (WB) |
| P35 | Thermo Fisher Scientific | MA5-14834 | 1/1000 (WB) |
| TPPP/P25 | Abcam | ab92305 | 1/1000 (WB) |
| β-actin | Proteintech | 66009-1 | 1/10000 (WB) |
| HRP-conjugated Goat anti-rabbit antibody | Beyotime | A0208 | 1/1000 (WB) |
| HRP-conjugated Goat anti-mouse antibody | Beyotime | A0216 | 1/1000 (WB) |

## RBPMS, RNA-binding protein with multiple splicing; Itgb1, integrin beta 1; Dab1, disabled 1; Isl1, Islet1; PKCα, protein kinase C alpha; PI3K, Phosphoinositide 3-kinase; Akt, protein kinase B; GSK3B, Glycogen Synthase Kinase 3 Beta; Bcl-2, B-cell lymphoma-2; Bax, Bcl-2 Associated X-protein; Caspase-3, cysteinyl aspartate specific proteinase 3; P35, CDK5 Regulatory Subunit; P25, Cleaved p35.

## **Table S2. Primer sequences used for the real-time quantitative reverse transcriptase polymerase chain reaction**

| **Species** | **Gene** | **Forward primer (5’-3’)** | **Reverse primer (5’-3’)** |
| --- | --- | --- | --- |
| Mouse | *Reln* | ATGGCAATGCTGTCACCTTCT | AGCGGTATTGTTCTTGGCGTA |
|  | *GAPDH* | CCTCGTCCCGTAGACAAAATG | TGAGGTCAATGAAGGGGTCGT |

*GAPDH,*[*glyceraldehyde*](https://baike.baidu.com/item/glyceraldehyde/24434816?fromModule=lemma_inlink)*-3-phosphate dehydrogenase*

**Figure Legends**

Figure S1.

Concentration selection of Reelin protein and the effect of alpelisib on mouse Body Weight. (A) Histopathological examination of retinal tissue stained with H&E showed the effect of different Reelin protein concentrations on the thickness of the inner retina 7 days after I/R injury. Treatment with Reelin protein at 300 ng/μl and 500 ng/μl significantly improved I/R induced internal retinal thinning. Scale bar: 80 μm. (B) Quantification of inner retinal thickness (n = 4). (C) Treatment with Alpelisib did not significantly influence the body weight of mice (n = 6). Data are represented as means ± SD. **P* < 0.05, ***P* < 0.01, ****P* < 0.001.

Figure S2.

Establishment of retinal I/R injury model and expression of *Reln* in the retina after I/R injury. (A) Representative images of 33-gauge needle injection into the mouse anterior chamber before and after I/R injury. (B) Representative fluorescent staining of retinal patches at different time points after I/R injury showed the RBPMS-positive RGCs (orange) distribution. The number of RGC decreased significantly due to I/R injury and stabilized after 7 days. Scale bars: 100 μm or 1mm. (C) Number of RBPMS-positive RGCs (n = 3). (D) Representative images of Reelin immunofluorescence staining at various time points after retinal I/R injury in wild-type mice. Scale bar: 40 μm. Data are represented as means ± SD. **P* < 0.05, ****P* < 0.001.

Figure S3.

Expression of *Reln* in CBCs and PPI network in *Reln* pathway. (A) t-SNE charts show the expression of the *Reln* gene in different retinal cell clusters in the blank and I/R groups, with the color representing the expression level. (B) PPI network display the interaction relationship between Reln, Itgb1, Dab1, PI3K and Akt. (C) The t-SNE map shows 45 distinct clusters of RGCs, each colored dot represents a cell. (D) Representative images of immunofluorescence staining of normal mouse retina, CBCs (ISL1- and Reelin-positive; PKCα-negative), and RBCs (ISL1- and PKCα-positive; Reelin-negative). Scale bar: 40 µm. (E) A heat map representing the scaled expression values of the top 10 genes defining each CBC-SC. (F) t-SNE distribution showing three SCs of CBCs. (G) t-SNE distribution showing CBCs in the blank and I/R groups. (H) The expression of the *Reln* gene in each CBC-SC of the blank and I/R groups.

Figure S4.

The apoptosis reached a peak at 24 hours after I/R injury. (A) Representative TUNEL staining of frozen retinal sections at different time points after I/R injury. Scale bar: 40 µm. (B) Quantification of TUNEL-positive cells (n = 3). Data are represented as means ± SD. ***P* < 0.01, *****P* < 0.0001.

Figure S5.

Expression of P35 and P25 after retinal I/R injury. (A) Representative blotting images of the effects of Reelin protein therapy on retinal P35 and P25 after I/R injury. (B) Quantitative analysis of the levels of the aforementioned proteins (n = 3). Data are represented as the means ± SD. ****P* < 0.001.
