# Supplementary figures and images for: Reln-Dab1 pathway mitigates retinal ganglion cell apoptosis in retinal ischemia-reperfusion injury
Source: Cell Death Dis. 2025 May 29;16(1):423. doi: 10.1038/s41419-025-07742-6 (PMC12122947; doi:10.1038/s41419-025-07742-6)

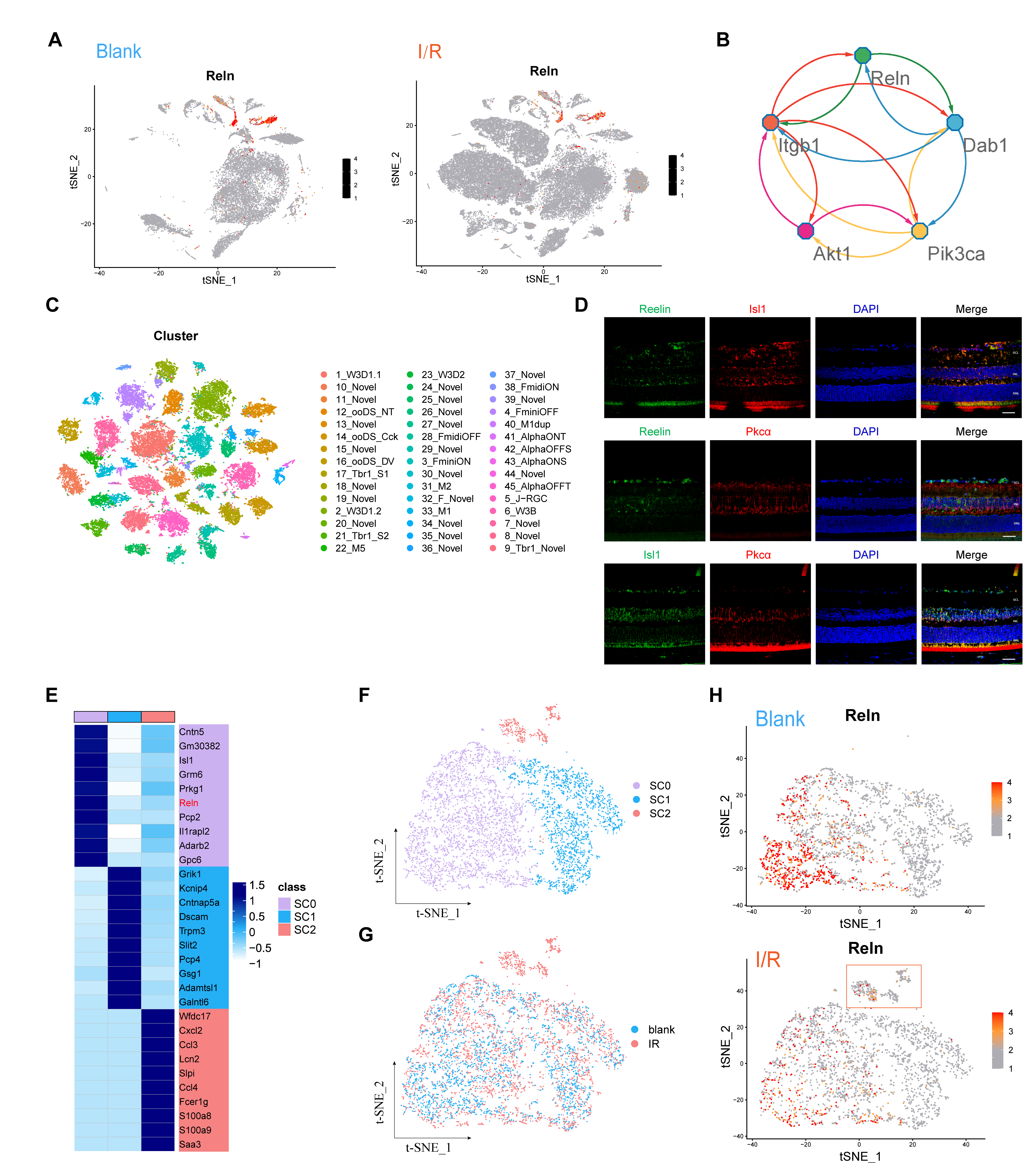

Supplement: Supplementary file 4 — figureS3 [file 41419_2025_7742_MOESM4_ESM.tif]

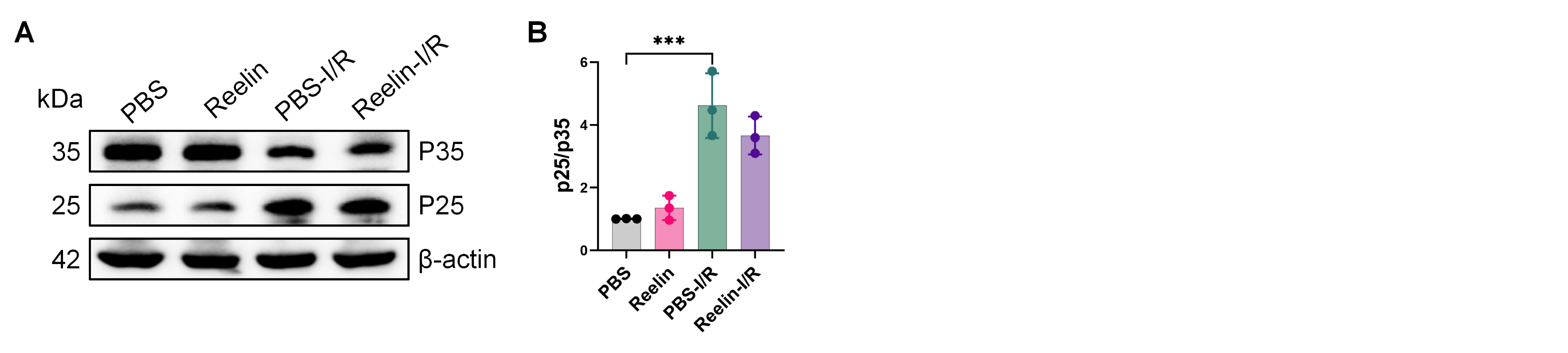

Supplement: Supplementary file 6 — Figure S5 [file 41419_2025_7742_MOESM6_ESM.tif]
