# Supplementary material for: Reln-Dab1 pathway mitigates retinal ganglion cell apoptosis in retinal ischemia-reperfusion injury
Source: Cell Death Dis. 2025 May 29;16(1):423. doi: 10.1038/s41419-025-07742-6 (PMC12122947; doi:10.1038/s41419-025-07742-6)
Supplement: Supplementary file 7 — Full and uncropped western blots [file 41419_2025_7742_MOESM7_ESM.pdf]

Uncropped/unedited images for all blots

The same protein molecular weight marker was used in all figures （26619；thermofisher)

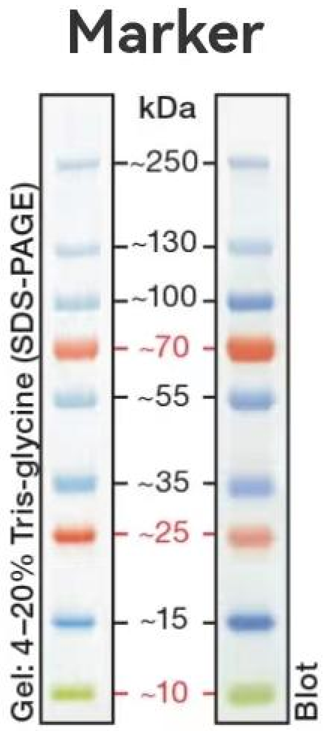

**fig 5C**

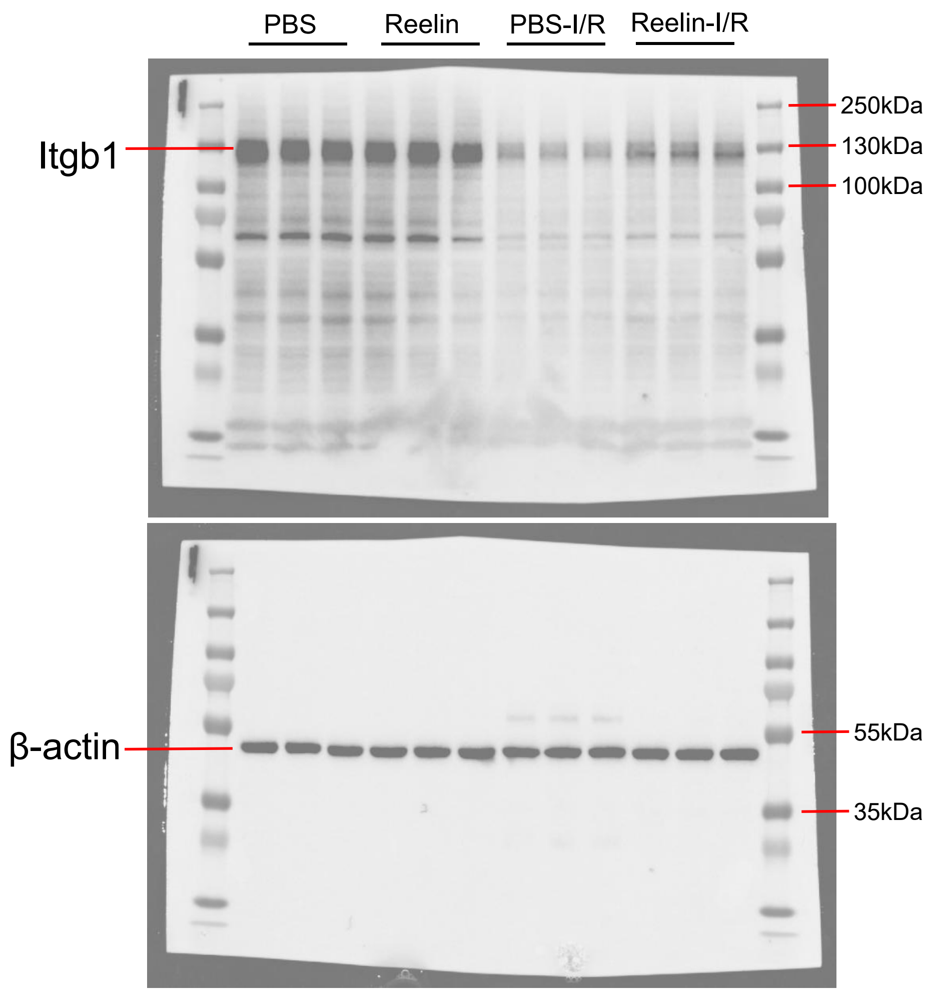

PBS
Reelin
PBS-I/R
Reelin-I/R
Itgb1
250kDa
130kDa
100kDa
β-actin
55kDa
35kDa


**fig 5D**

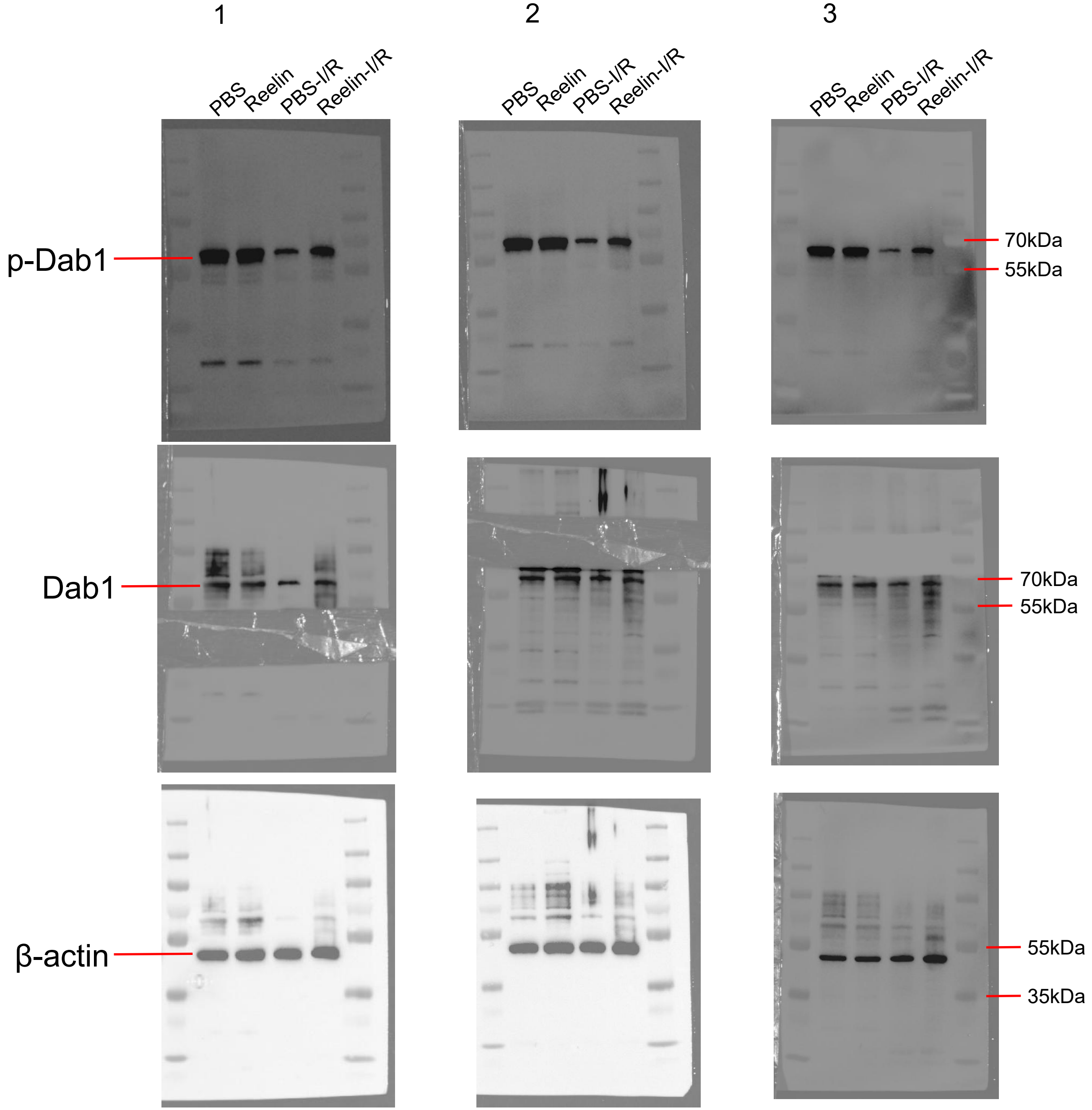

1
2
3
PBS
Reelin
PBS-I/R
Reelin-I/R
p-Dab1
Dab1
β-actin
70kDa
55kDa
70kDa
55kDa
55kDa
35kDa

**fig 5D**

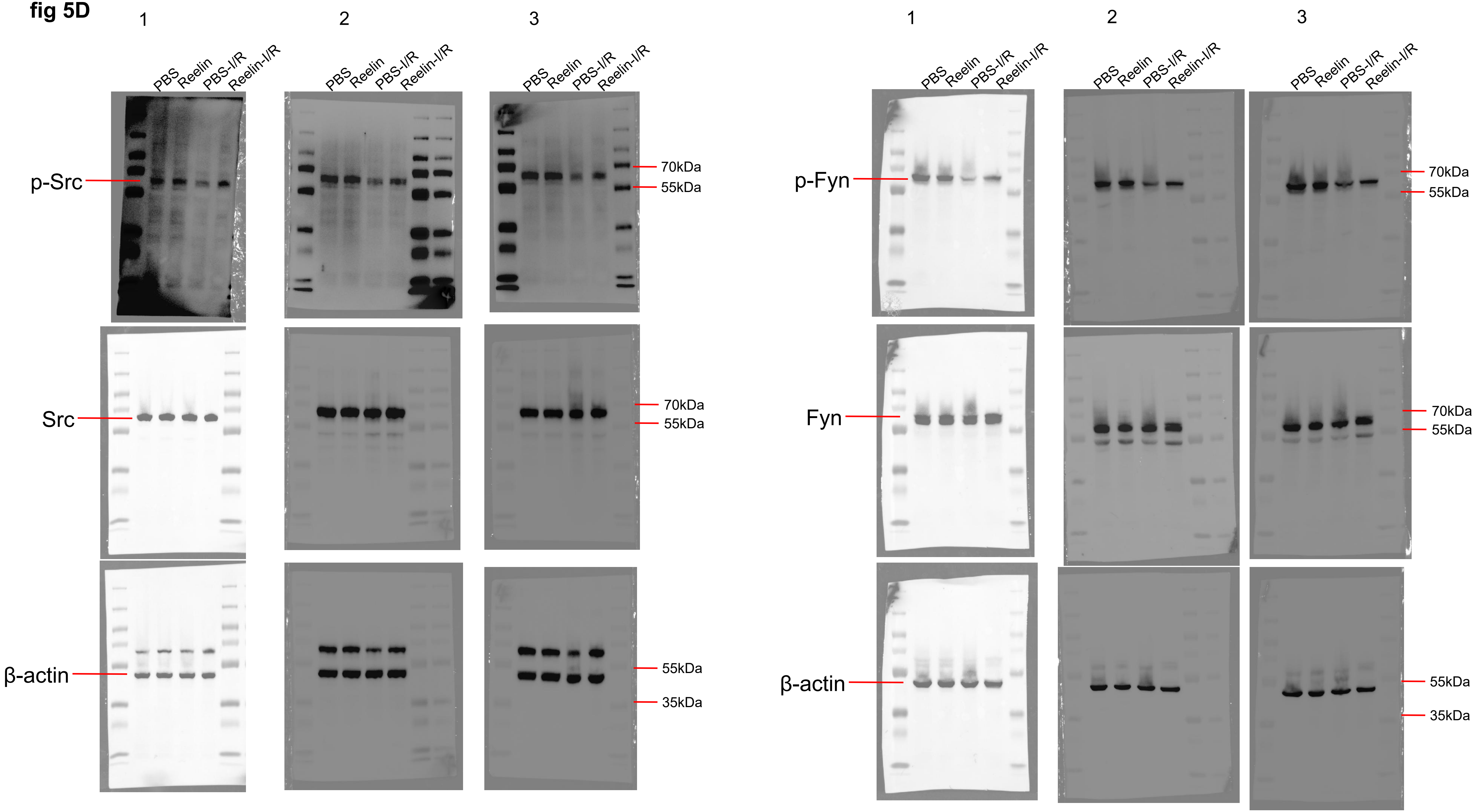

1
2
3
PBS
Reelin
PBS-I/R
Reelin-I/R
p-Src
Src
β-actin
70kDa
55kDa
35kDa
p-Fyn
Fyn

fig 5D

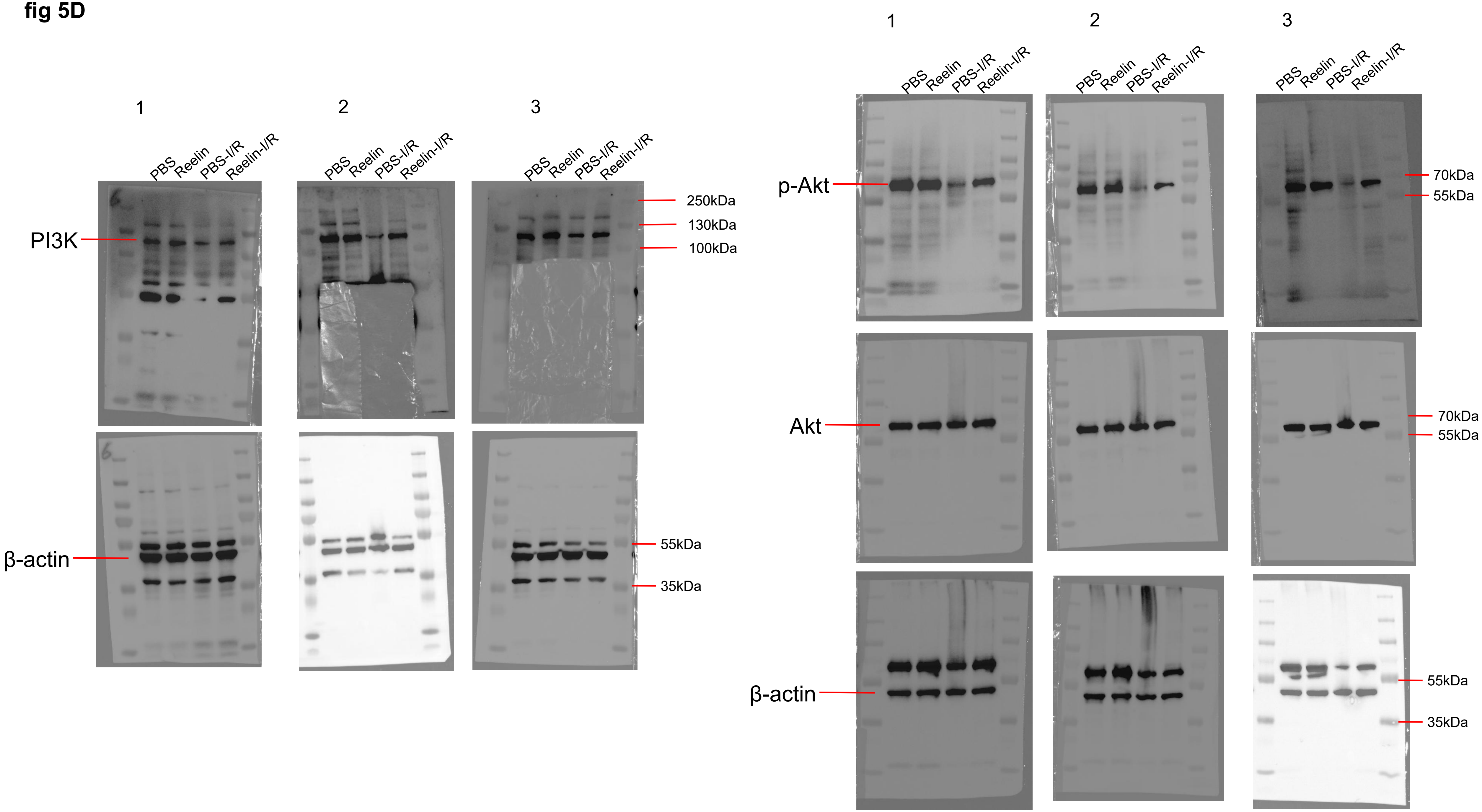

1
2
3
PBS
Reelin
PBS-I/R
Reelin-I/R
PI3K
250kDa
130kDa
100kDa
β-actin
55kDa
35kDa
p-Akt
70kDa
55kDa
Akt
70kDa
55kDa
β-actin
55kDa
35kDa

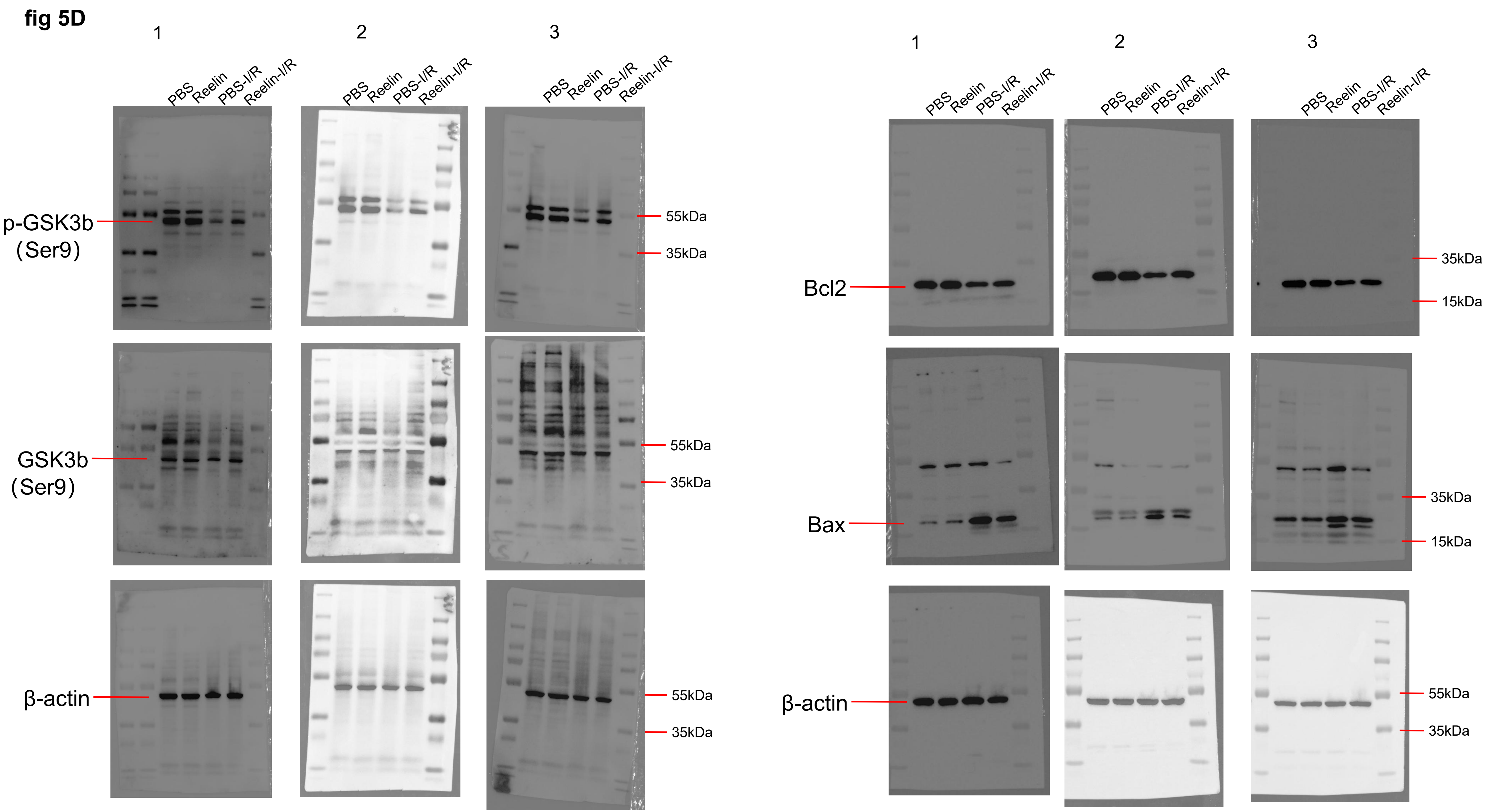
fig 5D
1
2
3
PBS
Reelin
PBS-I/R
Reelin-I/R
p-GSK3b (Ser9)
GSK3b (Ser9)
β-actin
55kDa
35kDa
Bcl2
Bax
15kDa

fig 5D

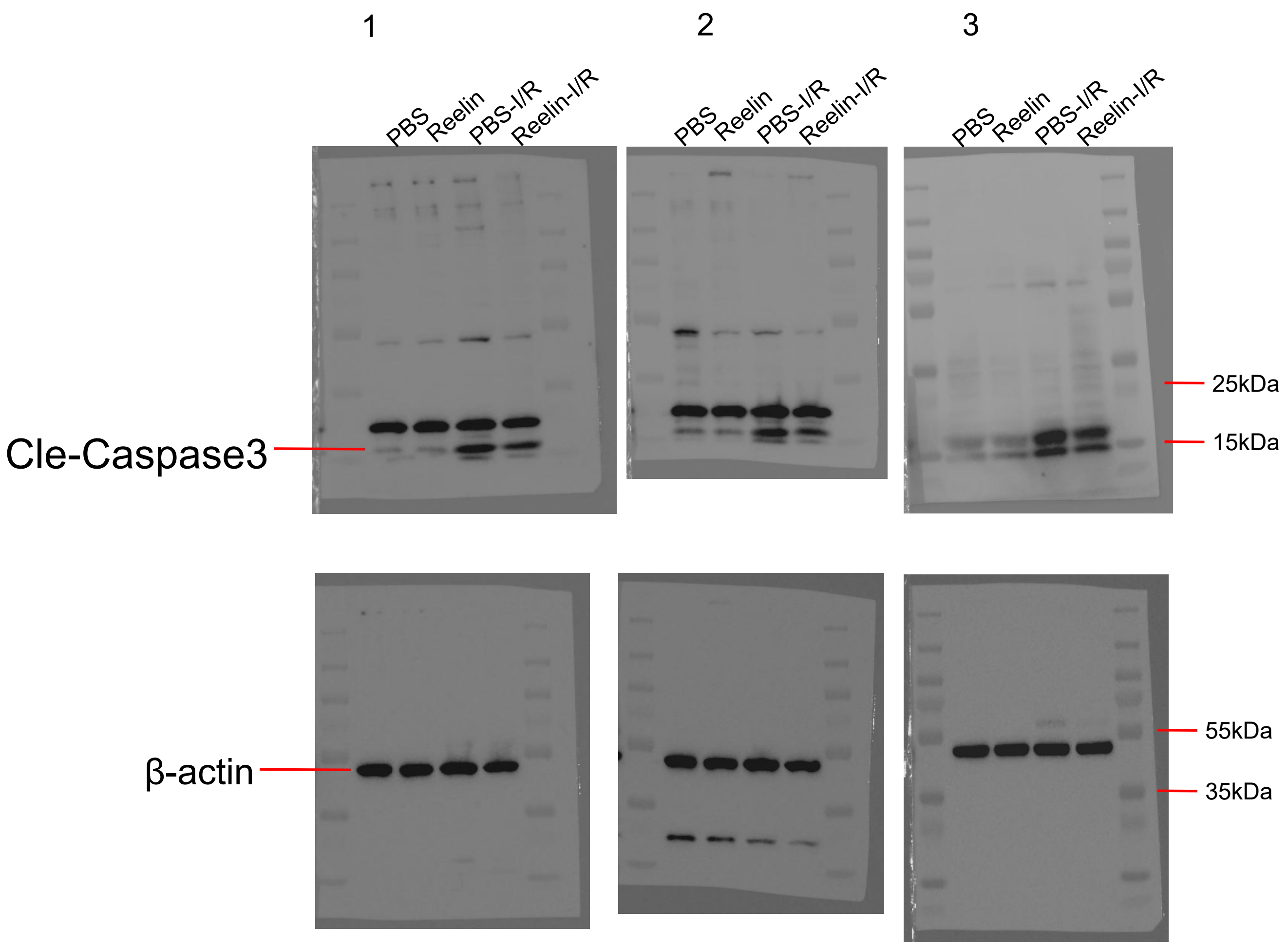

1
2
3
PBS
Reelin
PBS-I/R
Reelin-I/R
Cle-Caspase3
β-actin
25kDa
15kDa
55kDa
35kDa

**fig 5F**

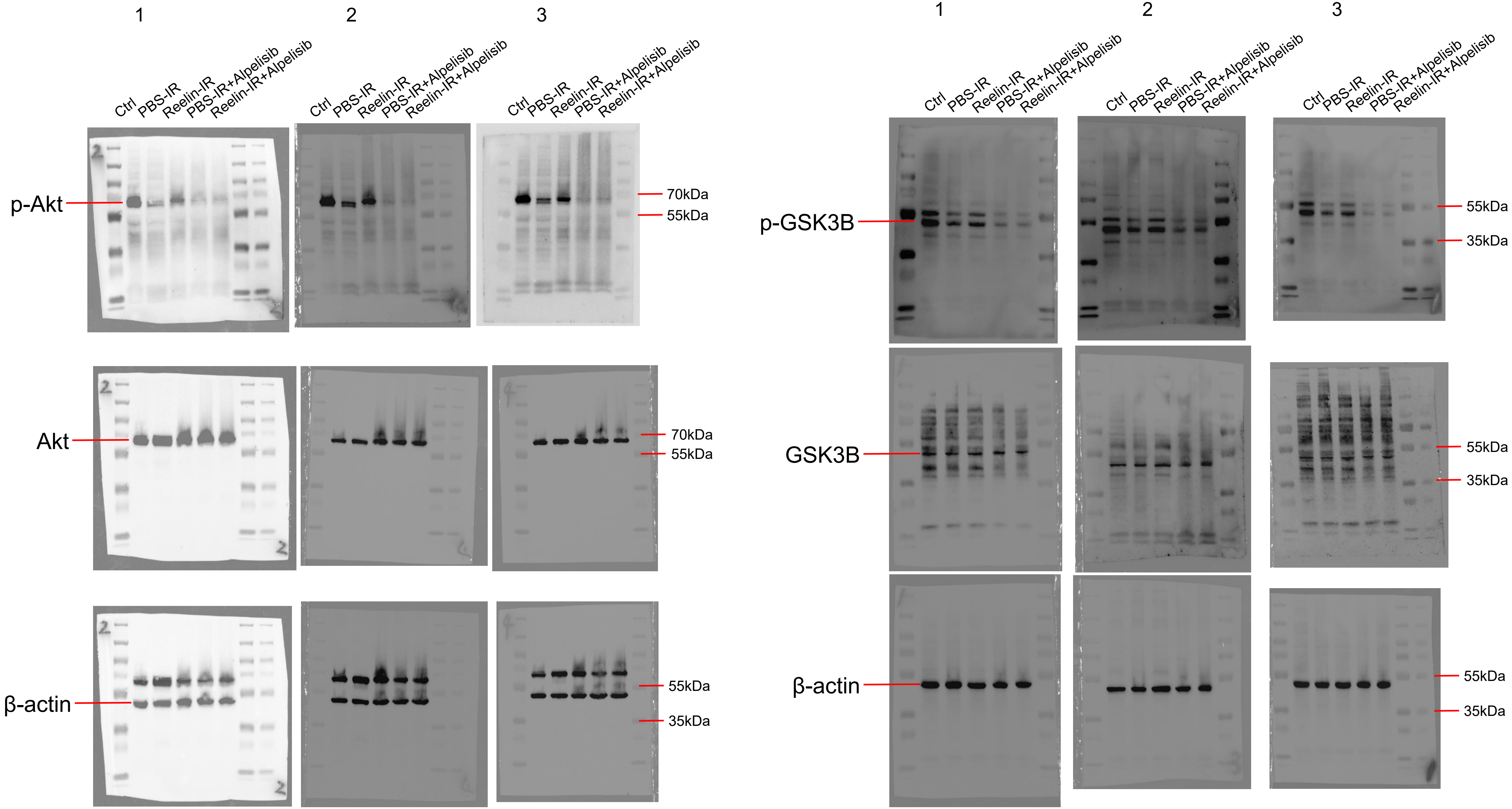
1
2
3
Ctrl
PBS-IR
Reelin-IR
PBS-IR+Alpelisib
Reelin-IR+Alpelisib
p-Akt
Akt
β-actin
70kDa
55kDa
35kDa
p-GSK3B
GSK3B

fig 5F

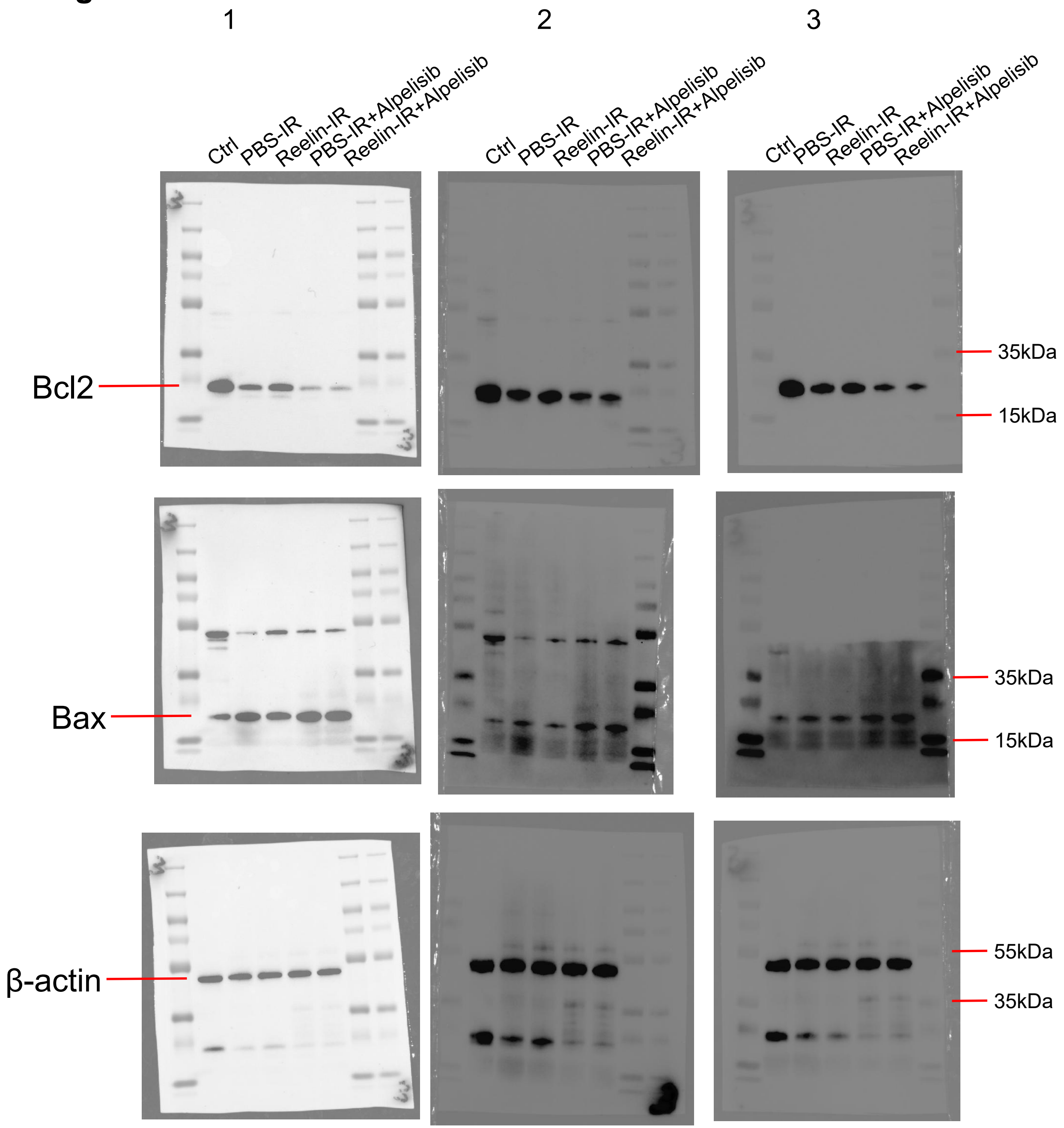

1
2
3
Ctrl
PBS-IR
Reelin-IR
PBS-IR+Alpelisib
Reelin-IR+Alpelisib
Bcl2
Bax
β-actin
35kDa
15kDa
55kDa
35kDa


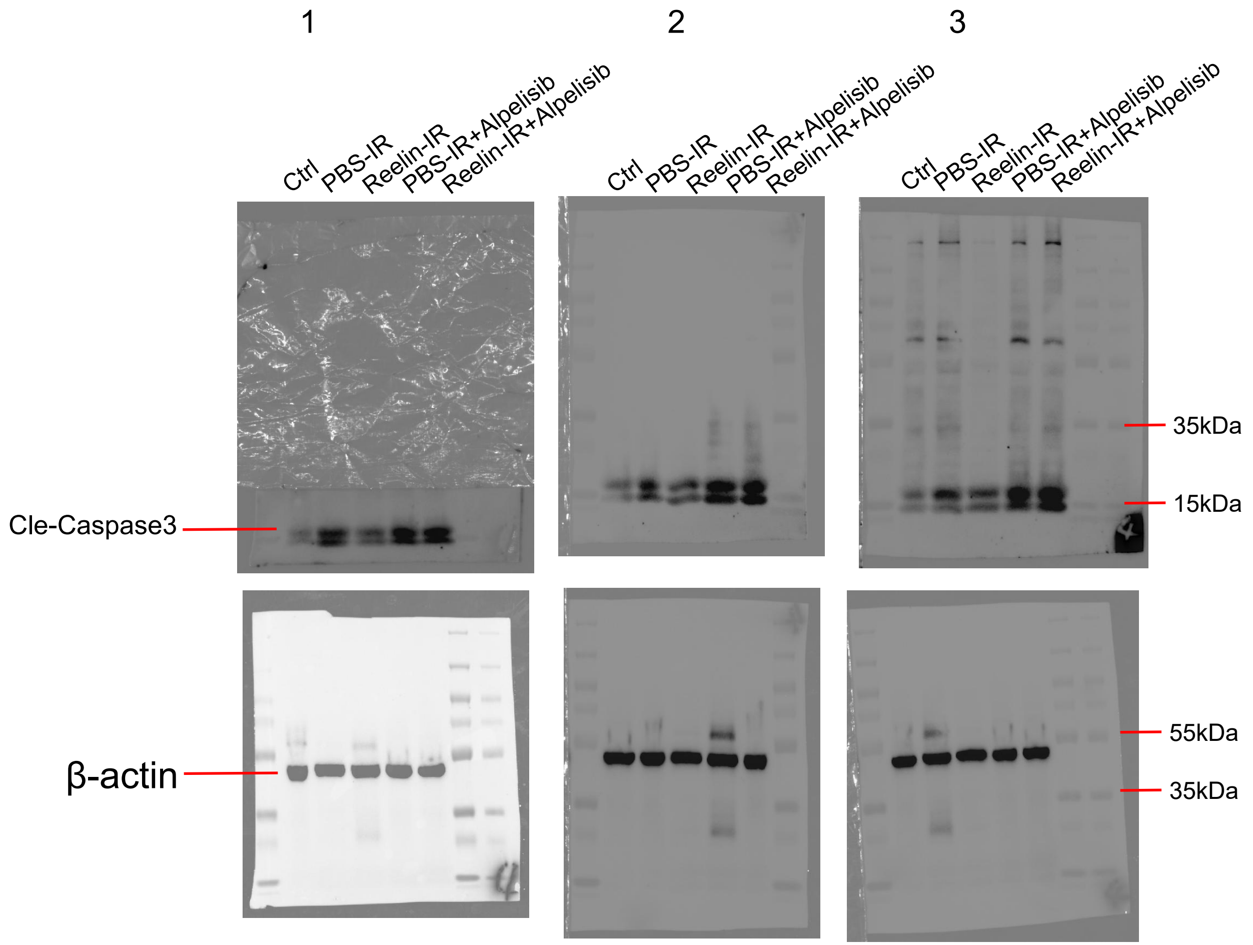

1
2
3
Ctrl
PBS-IR
Reelin-IR
PBS-IR+Alpelisib
Reelin-IR+Alpelisib
Cle-Caspase3
β-actin
35kDa
15kDa
55kDa
35kDa

fig 6C

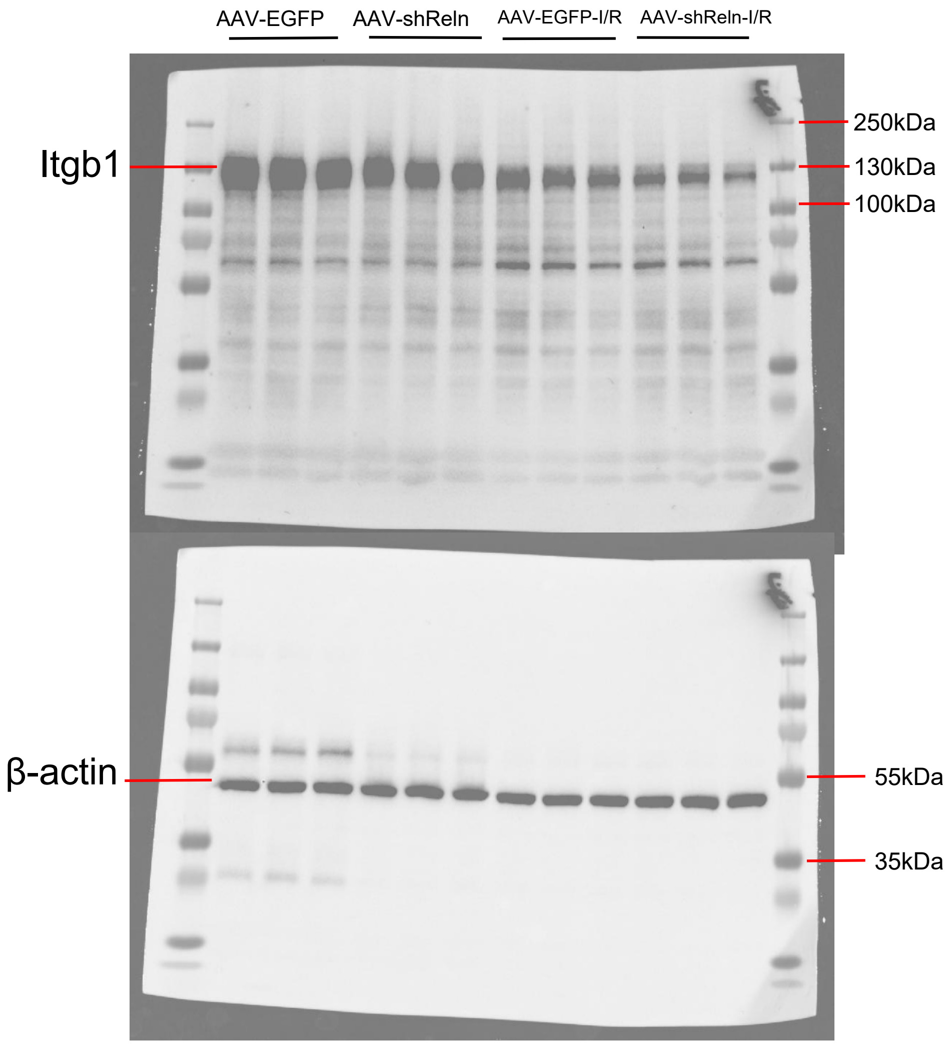
AAV-EGFP
AAV-shReln
AAV-EGFP-I/R
AAV-shReln-I/R
Itgb1
250kDa
130kDa
100kDa
β-actin
55kDa
35kDa

fig 6D

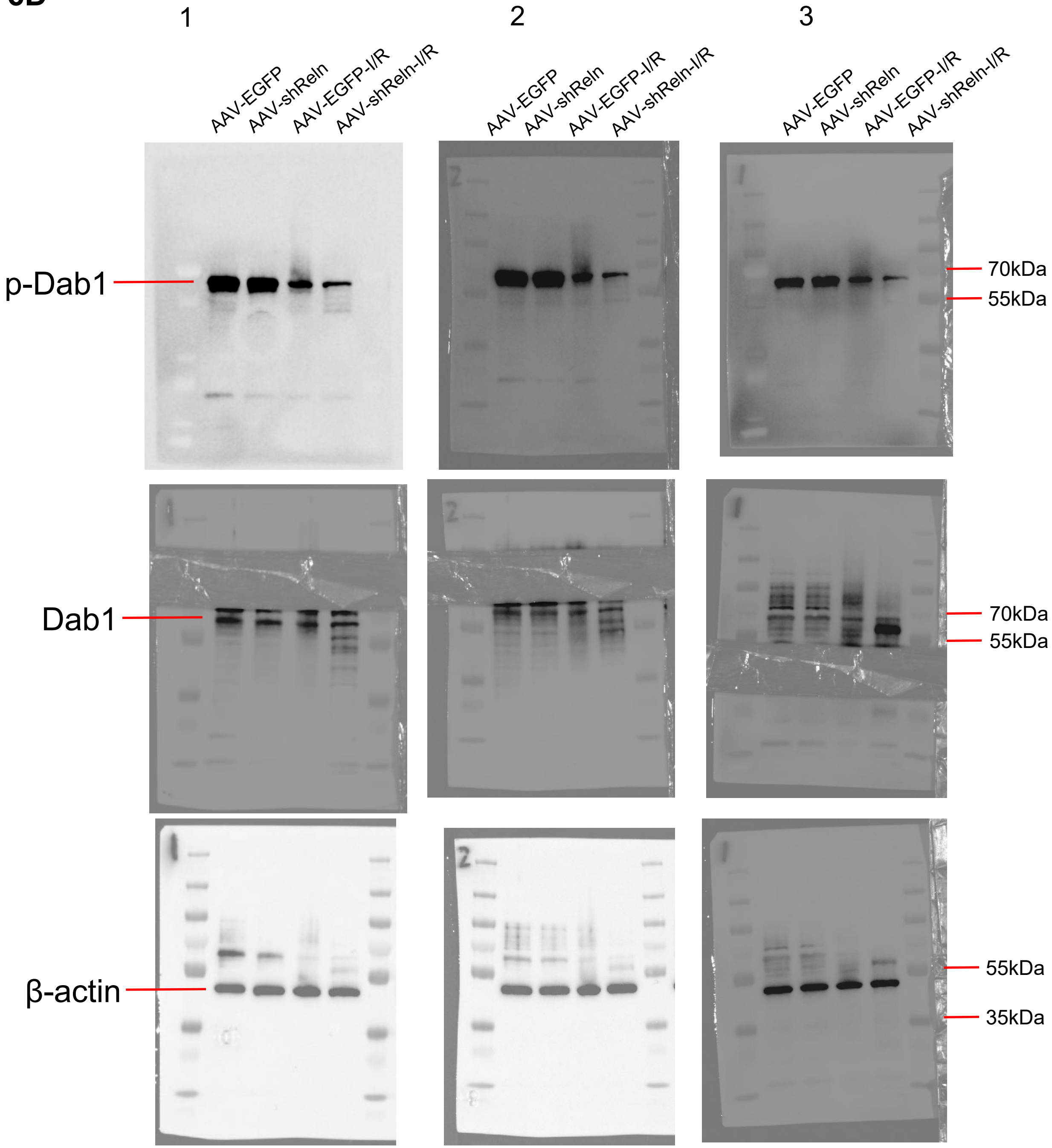
1
2
3
AAV-EGFP
AAV-shReln
AAV-EGFP-I/R
AAV-shReln-I/R
p-Dab1
70kDa
55kDa
Dab1
70kDa
55kDa
β-actin
55kDa
35kDa

fig 6D

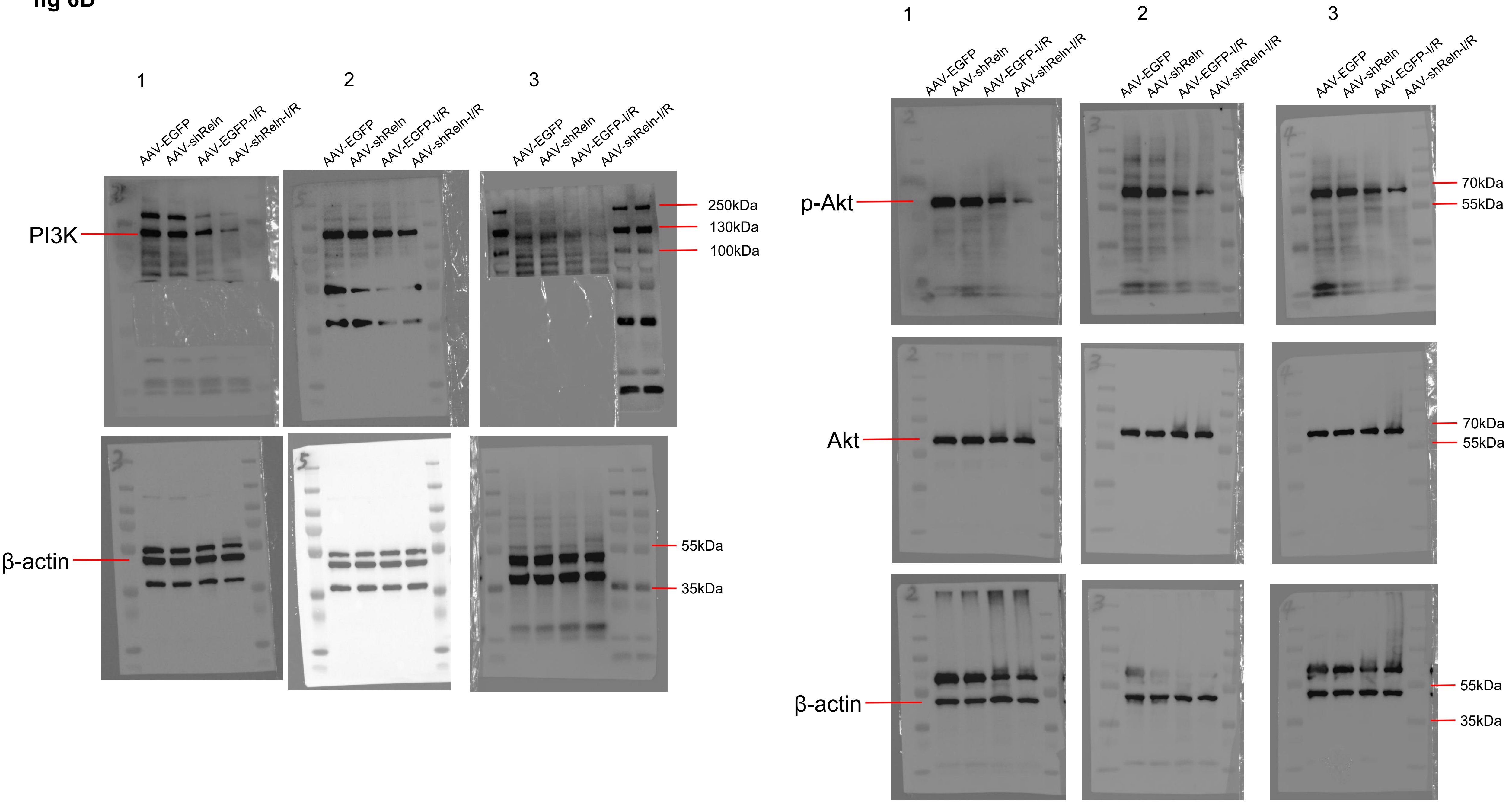
1
2
3
AAV-EGFP
AAV-shReln
AAV-EGFP-I/R
AAV-shReln-I/R
PI3K
β-actin
250kDa
130kDa
100kDa
55kDa
35kDa
p-Akt
Akt
70kDa

fig 6D

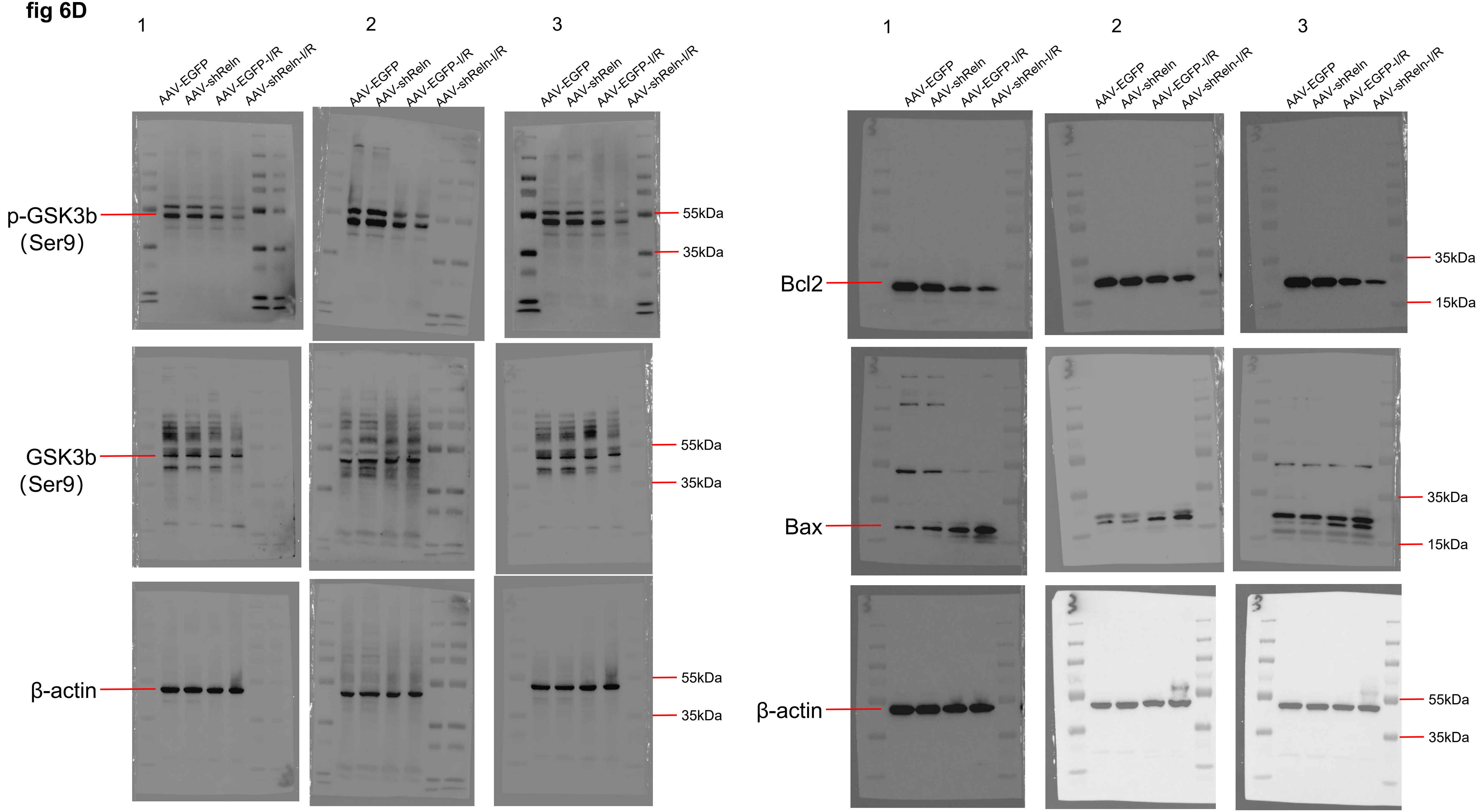
1
2
3
AAV-EGFP
AAV-shReln
AAV-EGFP-I/R
AAV-shReln-I/R
p-GSK3b (Ser9)
GSK3b (Ser9)
β-actin
55kDa
35kDa
Bcl2
Bax
15kDa

fig 6D

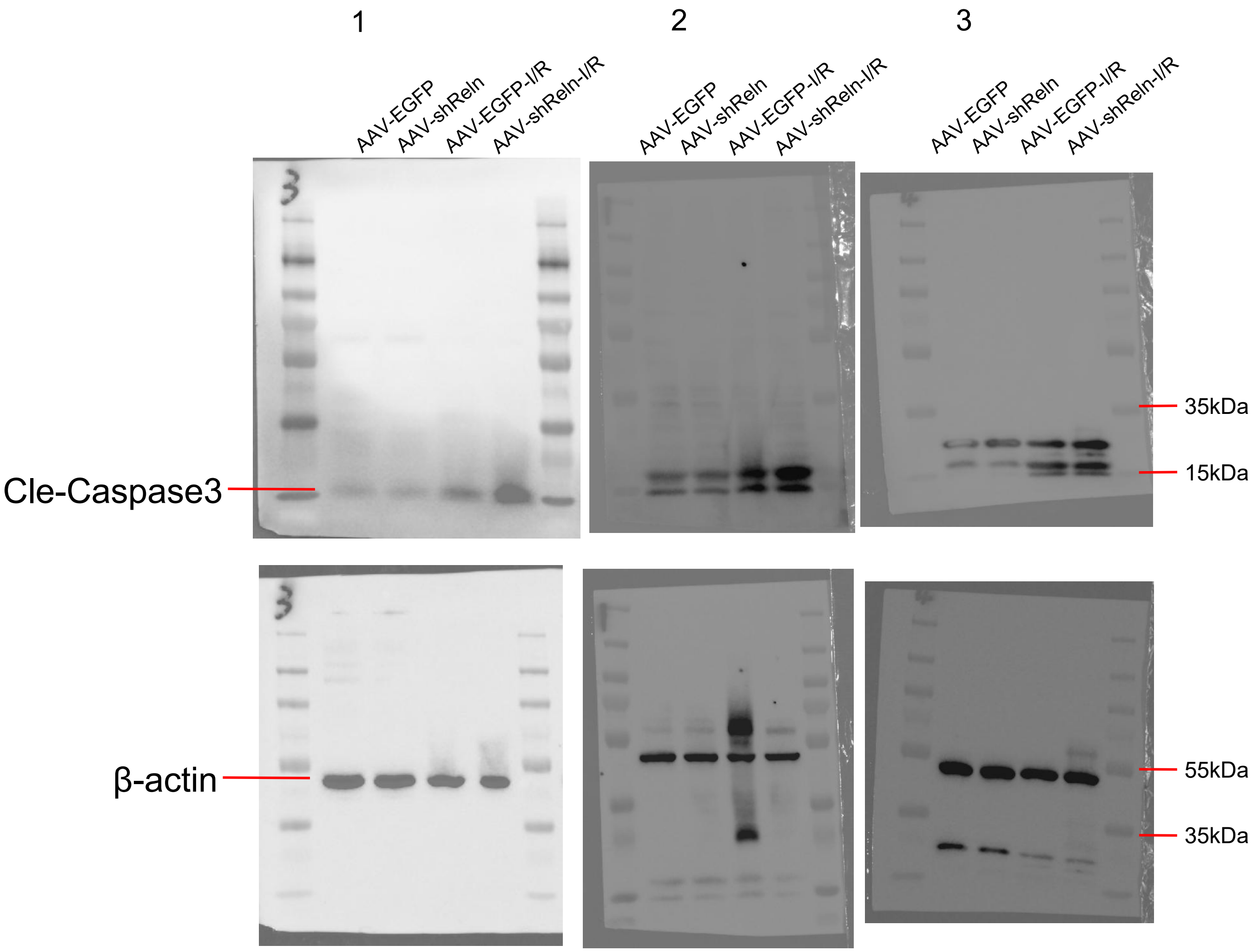
1
2
3
AAV-EGFP
AAV-shReln
AAV-EGFP-I/R
AAV-shReln-I/R
Cle-Caspase3
β-actin
35kDa
15kDa
55kDa
35kDa

fig S5A

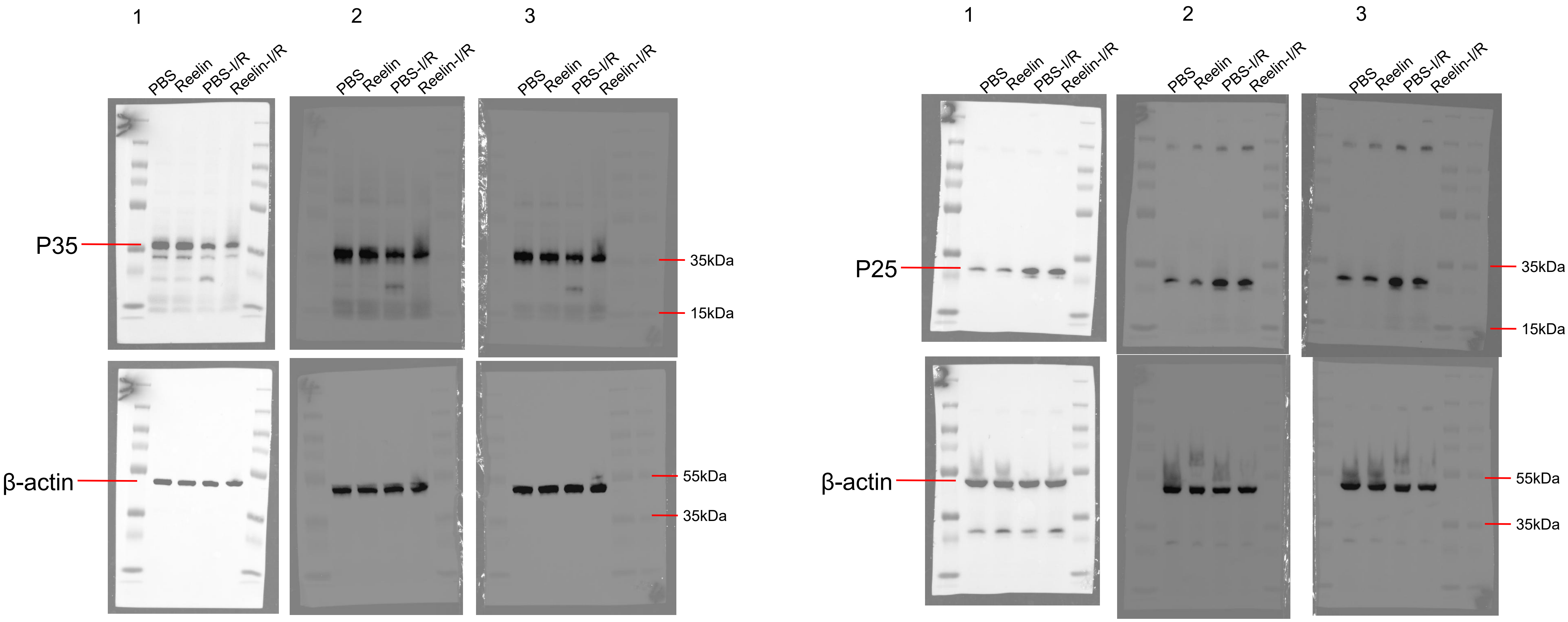
1
2
3
PBS
Reelin
PBS-I/R
Reelin-I/R
P35
35kDa
15kDa
β-actin
55kDa
35kDa
1
2
3
PBS
Reelin
PBS-I/R
Reelin-I/R
P25
35kDa
15kDa
β-actin
55kDa
35kDa
